# PEG-Like Nanoprobes: Multimodal, Pharmacokinetically and Optically Tunable Nanomaterials

**DOI:** 10.1371/journal.pone.0095406

**Published:** 2014-04-29

**Authors:** Yanyan Guo, Hushan Yuan, Natalie M. Claudio, Sreekanth Kura, Naomi Shakerdge, Thorsten R. Mempel, Brian J. Bacskai, Lee Josephson

**Affiliations:** 1 Center for Advanced Medical Imaging Sciences, Massachusetts General Hospital, Charlestown, Massachusetts, United States of America; 2 Center for Immunology and Inflammatory Diseases, Massachusetts General Hospital, Charlestown, Massachusetts, United States of America; 3 Martinos Center for Biomedical Imaging, Massachusetts General Hospital, Charlestown, Massachusetts, United States of America; 4 Alzheimer Research Unit, Department of Neurology, Massachusetts General Hospital and Harvard Medical School, Charlestown, Massachusetts, United States of America; Baker IDI Heart and Diabetes Institute, Australia

## Abstract

“PEG-like Nanoprobes” (PN’s) are pharmacokinetically and optically tunable nanomaterials whose disposition in biological systems can be determined by fluorescence or radioactivity. PN’s feature a unique design where a single PEG polymer surrounds a short fluorochrome and radiometal bearing peptide, and endows the resulting nanoprobe with pharmacokinetic control (based on molecular weight of the PEG selected) and optical tunability (based on the fluorochrome selected), while the chelate provides a radiolabeling option. PN’s were used to image brain capillary angiography (intravital 2-photon microscopy), tumor capillary permeability (intravital fluorescent microscopy), and the tumor enhanced permeability and retention (EPR) effect (^111^In-PN and SPECT). Clinical applications of PN’s include use as long blood half-life fluorochromes for intraoperative angiography, for measurements of capillary permeability in breast cancer lesions, and to image EPR by SPECT, for stratifying patient candidates for long-circulating nanomedicines that may utilize the EPR mechanism.

## Introduction

A design for multimodal, fluorescent/radioactive imaging agents embodying a versatile, simple, and clinically translateable platform has yet to be fully recognized. An ideal design would allow pharmacokinetic and optical tuning with a radiolabeling option, and be readily eliminated after providing diagnostic information, to lessen the chances of delayed side effects. Though a variety of novel nanomaterials have been described (e.g. nanoshells, carbon nanotubes, dendrimers, quantum dots), many suffer from a lack of clinical experience and a lack of understanding of their toxicity and elimination.

**Figure 1:**
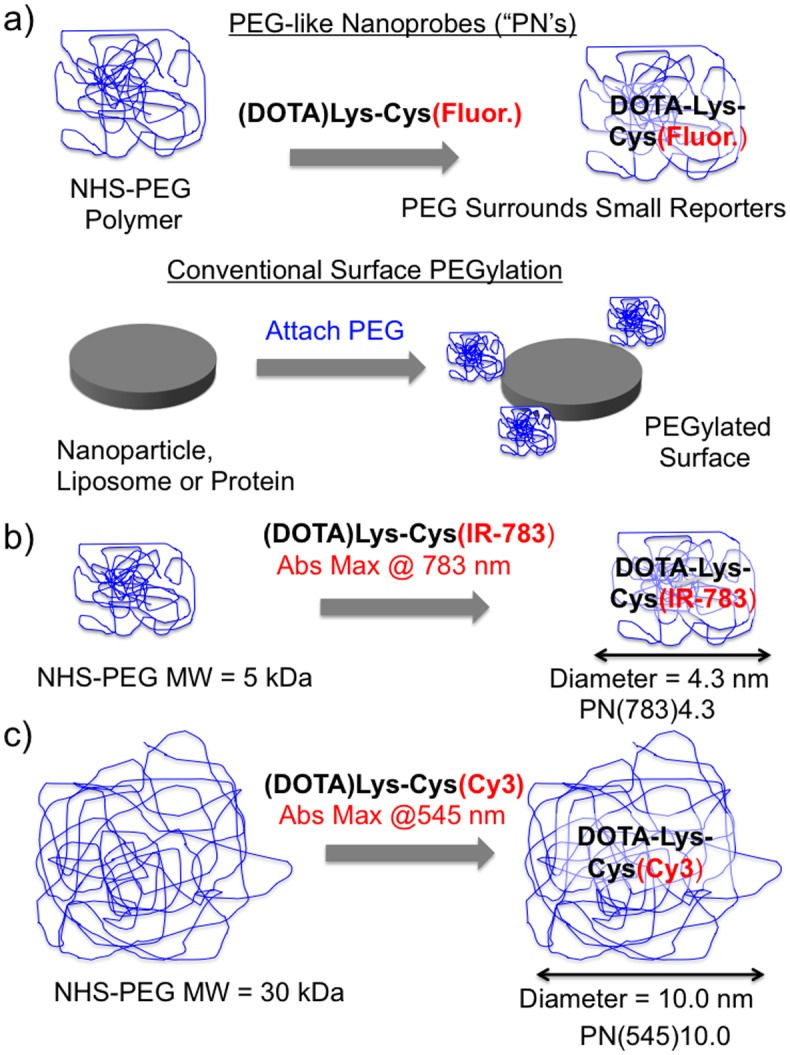
Reaction with PEG polymers and fluorochrome and DOTA bearing peptides yields PEG-like Nanoprobes (PN’s). **A**) Syntheses of PEG-like Nanoprobe (PN’s). The peptide (DOTA)Lys-Cys(Fluor.), with an N-terminal DOTA, a variable fluorochrome (Fluor.) attached to the cysteine side chain, and a single primary amine, reacts with the NHS ester of a variable PEG. In the conventional PEGylations of proteins or nanoparticles, multiple PEG’s provide a PEG bearing surface. This approach yields materials with a PEG to protein ratio or with a PEG surface density. **B**) Synthesis of the PN denoted PN(783)4.3. A 5 kDa PEG is reacted with the (DOTA)Lys-Cys(IR-783) peptide. The diameter (by FPLC) is 4.3 nm and the absorption maximum is 783 nm, hence PN(783)4.3. **C**) A 30 kDa PEG is reacted with the (DOTA)Lys-Cys(Cy3) peptide. The diameter is 10.0 nm and an absorption maximum is 545 nm, hence, PN(545)10.0. A list of PN’s and their properties is given in Table 1.

We introduce “PEG-like Nanoprobes” (PN’s) which employ a single PEG polymer to surround a central fluorochrome and chelate bearing peptide, a unique and clinically translateable design that confers pharmacokinetic and optical tunability on fluorescent and radioactive nanomaterials. PN’s exploit PEG-fluorochrome shielding [1] which has been used in the design of integrin binding RGD peptides, administered with peritumoral injection and which diffuse through the interstitium into a tumor, increasing tumor targeting and reducing normal organ uptake compared to that obtained with IV administration [2]. The use of a PEG in PN design is compared with the use of PEGylated in other nanomaterials [3]–[6] in **Figure 1A**. (Support for this configuration of PEG with PN’s is provided at end of the Discussion).

The PN design allows the pharmacokinetics of fluorochromes and radiometals to be optimized for specific applications by varying the size of the PEG, a widely available polymer recognized as safe after extensive clinical use [7]–[9]. Clinical applications of the passively targeted (pharmacokinetically targeted) PN’s described are discussed further below; these include intraoperative fluorescent angiography [10], fluorescent measurements of breast lesion capillary permeability, and imaging the enhanced permeability and retention (EPR) effect of tumors by SPECT. The EPR effect is thought to be important in the efficacy of long-circulating nanomedicines generally [11], [12], and specifically with doxil, a doxorubicin liposome [13], and with abraxane, a paclitaxel/albumin complex [14]. Imaging tumor EPR effects might serve to select patients most likely to benefit from nanomedicines.

## Results

### 1. Synthesis and Characterization

To demonstrate the optical and pharmacokinetic tunability of PEG-like Nanoprobes, we synthesized PN’s using different fluorochromes, and different PEG polymers, as shown in **Figures 1B and 1C**. After synthesis of the N-terminal DOTA-bearing peptide, a thiol reactive fluorochrome is reacted with cysteine thiol, followed reaction of a PEG-NHS ester with lysine side chain. Details of the synthesis are provided in **Schemes S1-S7** in File S1. All peptides are C-terminal amides with the amide not shown in Figure 1 because, unlike the N-terminal and lysine side chain nitrogens, the C-terminal amide is not chemically reactive. The use of the IR-783 fluorochrome and 5 kDa PEG (**Figure 1B**) yields a PN with an absorption maxima of 783 nm and diameter of 4.3 nm; this is denoted as PN(783)4.3. The use of a Cy3 fluorochrome and a 30 kDa PEG (**Figure 1C**) yields a PN with an absorption maxima of 545 nm and a diameter of 10.0 nm; this is denoted as PN(545)10.0. Table 1 summarizes PN’s made in PN nomenclature (column 1) or peptide nomenclature (column 2). PN’s were synthesized with IR-783, Cy3 and fluorescein, with PEG’s ranging from 2 kDa to 40 kDa. Purification and characterization of PN’s is shown in **Figure S1** in File S1.

**Table 1:** Summary of PEG-like Nanoprobes (PN’s).

PNDesignation	PeptideDesignation	Abs/em(nm)	MW,(kDa) Obs*	Volume FPLC Dia. (Fig. 2a)**	Quant. Yield(Fig. S3)***	Cell NSB(Fig. S4)***
*Not Appl.*	*(DOTA)Lys-Cys*	*Not Appl.*	*0.6356*	*Not Appl.*	*Not Appl.*	*Not Appl.*
Not Appl.	(DOTA)Lys-Cys(IR-783)	789/807	1.3258	Not Appl.	0.053±0.008	95±1%
PN(783)3.0	(DOTA)Lys(PEG 2 kDa)-Cys(IR-783)	789/810	3.34	11.2 kDa 3.0 nm	0.13±0.02	50±1%
PN(783)4.3	(DOTA)Lys(PEG 5 kDa)-Cys(IR-783)	789/810	6.29	35.2 kDa 4.3 nm	0.16±0.03	20±1%
PN(783)6.1	(DOTA)Lys(PEG 10 kDa)-Cys(IR-783)	789/810	11.41	100.7 kDa 6.1 nm	0.16±0.03	14±1%
PN(783)8.4	(DOTA)Lys(PEG 20 kDa)-Cys(IR 783)	789/810	21.28	262.5 kDa 8.4 nm	0.16±0.04	14±1%
PN(783)10.0	(DOTA)Lys(PEG 30 kDa)-Cys(IR-783)	789/810	30.62	435.4 kDa 10.0 nm	0.16±0.04	18±1%
PN(783)11.7	(DOTA)Lys(PEG40 kDa)-Cys(IR-783)	789/809	45.00	690 kDa 11.7 nm	0.16±0.02	17±1%
*Not Appl.*	*(DOTA)Lys-Cys(Cy3)*	*544/559*	*1.2138*	*Not Appl.*	*0.32±0.07*	*98±1%*
*PN(545)4.3*	*(DOTA)Lys(PEG 5 kDa)-Cys(Cy3)*	*545/560*	*6.18*	*35 kDa*	*0.59±0.09*	*31±1%*
*PN(545)10.0*	*(DOTA)Lys(PEG 30 kDa)-Cys(Cy3)*	*545/560*	*30.51*	*435 kDa*	*0.61±0.10*	*15±1%*
Not Appl.	(DOTA)Lys-Cys(Fl)	497/521	1.0626	Not Appl.	0.50±0.11	0.4±0.1%
PN(497)4.3	(DOTA)Lys(PEG 5 kDa)-Cys(Fl)	497/521	6.04	35 kDa	0.59±0.06	0.2±0.1%
PN(497)10.0	(DOTA)Lys(PEG 30 kDa)-Cys(Fl)	497/521	30.36	435 kDa	0.60±0.08	0.1% ±0.1%

*M.W. Obs. = molecular weights were determined from mass spectrometry results.

**Volumes are expressed as diameters in nm (to enable comparison with nanomaterials) and as the equivalent volumes of proteins (to enable comparison with proteins).

***Values are means±1 S.D.

We determined the molecular weights of PN’s from mass spectroscopy **(** **Table 1** **, column 4)**. PN molecular weights reported are the sum of its components: the PEG polymer, and the peptide bearing a chelate and fluorochrome, i.e., the (DOTA)Lys-Cys(Fl) peptide, where Fl indicates IR-783, Cy3 or fluorescein. For example, as shown in Table 1, PN(783)4.3 has a molecular weight of 6.29 kDa which results from the 1.3258 kDa (DOTA)Lys-Cys(IR-783) peptide and the 5 kDa PEG.

**Figure 2:**
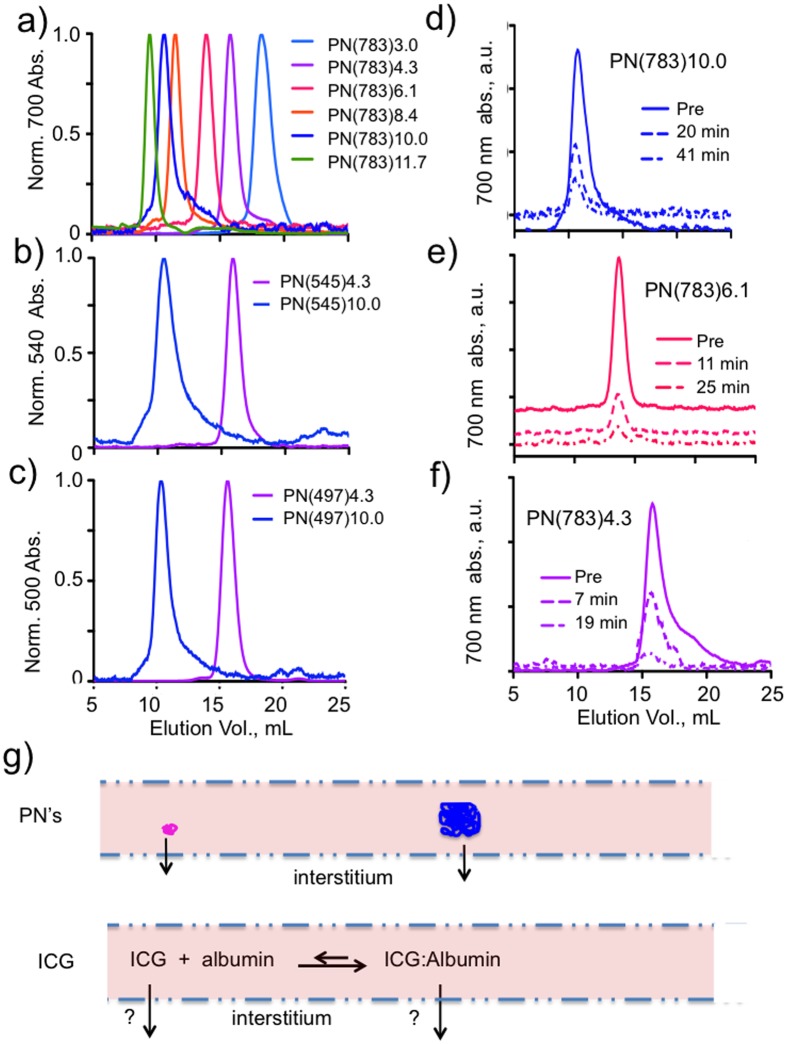
Tuning PN size, optical properties and PN circulating form. **A**) FPLC chromatograms of size-variable PN’s (constant IR-783 fluorochrome, variable PEG’s) are shown. Diameters are given in Table 1. **B,C**) Tuning PN size with different fluorochromes. The 5 kDa PEG yielded the magenta coded chromatograms with diameters of 4.3 nm regardless of the fluorochrome used. They are PN(783)4.3 (**2A**), PN(545)4.3 (**2B**), and PN(497)4.3 (**2C**). The 30 kDa PEG yielded the blue coded PN’s of 10 nm. Therefore PN dimensions are determined by PEG and independent of the fluorochrome selected. (**D,E,F**) FPLC chromatograms of PN’s are shown before (pre) and from the sera of injected mice. PN’s were PN(783)10.0 (**2D**), PN(783) 6.1 (**2E**), and PN(783)4.3 (**2F**). After injection, PN’s circulate at their PEG-determined and variable pre-injection sizes. (**G**) Since PN’s circulate at PEG-determined, pre-injection sizes, they cross capillaries at those sizes. ICG binds albumin and can cross capillaries in two forms. “FL” is the fluorescein fluorochrome.

To determine the volumes of PN’s in solution, we performed the FPLC experiments shown in **Figure 2**. FPLC chromatograms are shown for PN’s bearing the IR-783 fluorochrome with PEG’s ranging from 2 to 40 kDa (**Figure 2A**). FPLC was calibrated with globular protein standards, and globular protein equivalent molecular volumes (in kDa) were obtained (**Table 1** **,** column 5). Next, these globular protein equivalent molecular volumes were converted to PN diameters in nm using the relationship: 
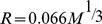
 where M is the molecular weight of a globular protein expressed in daltons [15]. PN diameters ranged from 3.0 to 11.7 nm, which means PN’s can be smaller or larger than albumin (MW = 67 kDa; diameter = 5.4 nm, by the formula above). Thus Table 1 column 5 gives the PN volumes obtained from FPLC expressed in two ways: (i) as the molecular weight in kDa of globular proteins with equivalent volumes, permitting size comparison of PN’s to proteins, and, (ii) as spherical diameters (“Dia.”) in nm, permitting size comparisons of a PN to relative to other nanomaterials. We confirmed the sizes obtained with FPLC by measuring the size of PN(783)11.7 by dynamic light scattering (11.7 nm). See **Figure S3** in File S1. Smaller PN’s did not scatter sufficient light for accurate size determination by this technique.

An analysis of PN composition indicates their PEG component largely determines both the nanoprobe’s composition and volume. Consider PN(783)4.3 from **Table 1** **,** columns 4 and 5. This PN consists of the (DOTA)Lys-Cys(IR783) (MW = 1.3258 kDa) peptide and a PEG (MW = 5 kDa), giving it a composition that is 79% PEG, (5000 Da/6290 Da). On a volume basis, the globular equivalent protein volume of this PN was 35.2 kDa (corresponding to a sphere with a diameter of 4.3 nm), which is a far larger volume than would be expected based on nanoprobe mass (6.29 kDa). Others have shown that PEG’s are extended, water infiltrated structures whose dimensions in solution are bigger than might be indicated from their mass [16]. Thus PN’s consist of a relatively long PEG polymer which provides most of nanoprobe mass and volume, and which surrounds a chelate and fluorochrome-bearing peptide as shown in **Figure 1A**. (Evidence for PEG on the outside and peptide on the inside is summarized in the end of the Discussion section below.) In contrast many PEGylated proteins and liposomes are obtained by attaching multiple PEG’s to the surfaces of starting materials, **Figure 1A**.

Linking the fluorochrome and the PEG significantly increased the quantum yields of fluorochrome-bearing peptides as summarized in **Table 1** **(column 6)**. (The p values showing significances are in **Figure S2** in File S1). The attachment of a 2 kDa PEG to (DOTA)Lys-Cys(IR-783) (i.e. yielding PN(783)3.0), resulted in significantly less improvement in quantum yield than larger PEG polymers; this was scored as “incomplete PEG-fluorochrome shielding” and only PN’s made with PEG’s of 5 kDa or greater were studied in biological systems. PEG attachment increased quantum yields in all cases, but as discussed below the magnitude of PEG generated improvement varied with the fluorochrome chosen. A mechanism for the PEG improvement of quantum yield is fluorochrome shielding, or the blocking of fluorochrome-fluorochrome interactions that result in quenching [1].

We next examined the non-specific binding (NSB) of PN’s to HT-29 cells based on the assumption that NSB minimization will correlate with a desirable minimization of post-IV injection retention. As noted above, after diagnostic drugs generate their added information, retention can only increase the risks of delayed toxicity. The attachment of PEG decreased the non-specific binding (NSB) to HT-29 cells, scored as the percent of cells above the cutoff for unstained cells seen with FACS **(** **Table 1** **, column 7)**. The FACS analyses used to measure NSB’s are provided in **Figure S4** in File S1. As with quantum yield, reduction in cell NSB is highly fluorochrome dependent, with the IR-783 peptide showing a high NSB, and profound reduction by PEG attachment, while the fluorescein peptide had a low NSB, even without PEG.

To examine whether the PN size was independent of the fluorochrome selected, PN’s made with IR-783, Cy3 and fluorescein were analyzed by FPLC (**Figures 2A, 2B, 2C**). With the 5 kDa PEG, PN diameters were 4.3 nm (magenta chromatograms of **Figures 2A–2C**) for PN’s with each fluorochrome. With the 30 kDa PEG and these fluorochromes (blue chromatograms, **Figures 2A–2C**), PN diameters were now 10.0 nm. Thus, the PEG we selected determined PN size, regardless of fluorochrome used.

To see if the variable PN size could be translated into tunable pharmacokinetics, it was essential to first establish that PN’s circulated at PEG-determined, pre-injection dimensions. Mice were injected with PN’s and FPLC chromatograms of serum samples, taken at various times post injection (**Figures 2D, 2E and 2F**), compared with pre-injection PN samples. By using PN’s absorbing at 783 nm, chromatograms from the sera of injected mice reflect this PN, since there are no compounds in serum that absorb at this wavelength. With the FPLC chromatograms of PN(783)10.0 (**Figure 2D**), peaks from a pre-injection sample or from serum samples at 21 or 40 minutes had identical peak elution volumes, indicating this PN circulates at its pre-injection size. Similarly, peaks for PN(783)6.1 (**Figure 2E**) and PN(783)4.3 (**Figure 2F**) where identical for pre-injection samples and for nanoprobes in the sera of injected mice. Thus, molecular weight of the PEG chosen determines PN dimensions in vitro and those dimensions are maintained after injection.

Since PN’s circulate at PEG-determined sizes, they are size-variable nanomaterials that undergo transcapillary passage as their injected form, see **Figure 2G**. In contrast, albumin-binding compounds like indocyanine green (ICG), used clinically to determine transcapillary passage (below), exist as albumin bound and free forms. PN’s are size variable, multimodal nanomaterials that can be used for determination of capillary permeability without the uncertainties presented by albumin bound and free forms.

**Figure 3:**
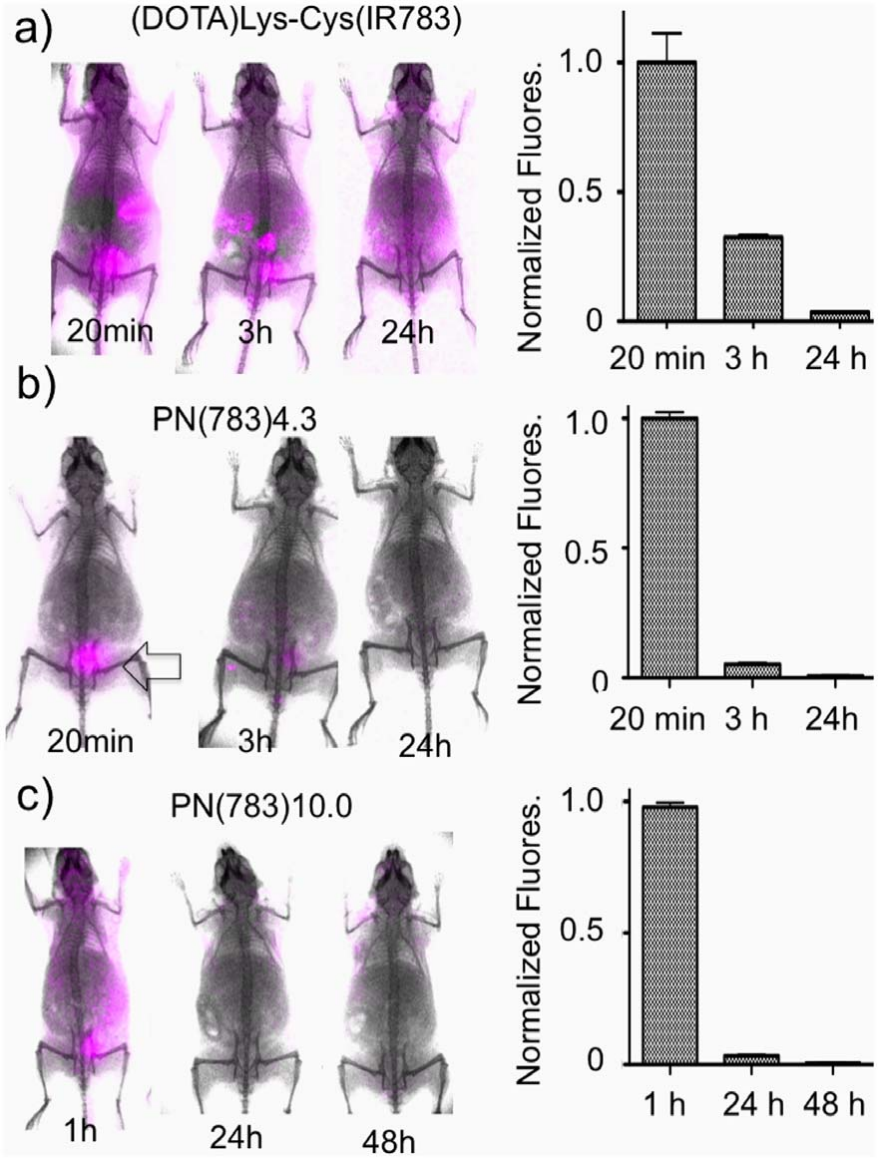
Role PEG in determining elimination by surface fluorescence imaging. **A**) Animals were injected with unPEGylated peptide and imaged at the times indicated. Bar graph gives whole animal surface fluorescence determined as means and standard deviations, n = 6. **B**) With the 5 kDa PEG attached, PN(783)4.3 is obtained, which shows renal elimination within 20 minutes (arrow). **C**) With the 30 kDa PEG, PN(783)10.0 is obtained, which shows a long vascular phase followed by elimination. Biodistribution and elimination of ^111^In-PN(783)10.0 is shown in Figure 6.

The effect of PEG on PN elimination from surface fluorescence measurements is shown in **Figure 3**. Surface fluorescence images for representative animals are shown and total surface fluorescence is quantified in bar graphs to the right of the image. A non-PEG bearing (DOTA)Lys-Cys(IR-783) peptide showed slow and incomplete elimination (**Figure 3A**), while PN(783)4.3, (**Figure 3B**), a PN with a 5 kDa PEG attached, showed bladder accumulation (arrow) at only 20 minutes post injection. PN(783)10.0, which was larger than albumin with a diameter of 5.4 nm showed a vascular phase at 1 h, with excellent elimination at 48 h. The pharmacokinetics and biodistributions of these PN’s is further analyzed below.

### 2. Pharmacokinetic Modeling and Pharmacokinetic Control

**Figure 4:**
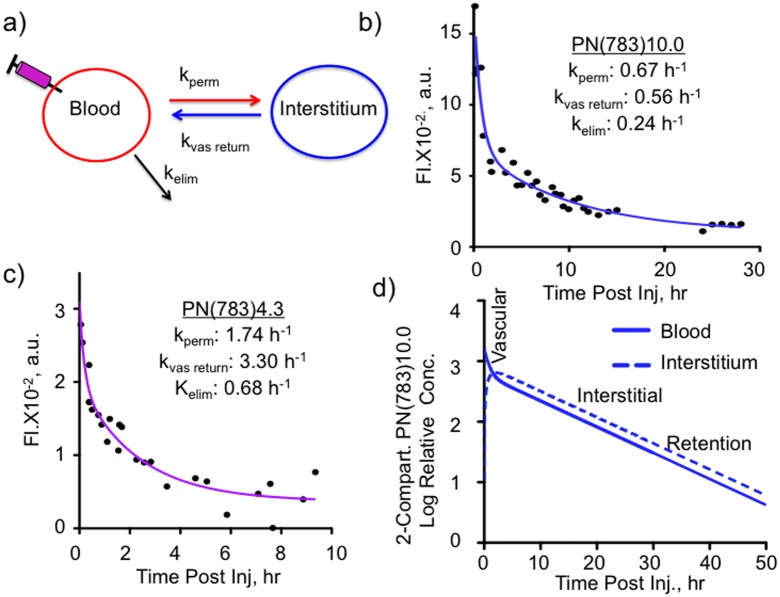
Variable PN pharmacokinetics analyzed by the two compartment pharmacokinetic model in normal mice. **A**) Two compartment pharmacokinetic model showing three microscopic rate constants. Serum fluorescence for PN(783)10.0 **B**) and PN(783)4.3 **C**) after injection are shown. Data were fit to the two compartment model with data provided in Table 1. **D)** Time courses for blood and interstitial fluorescence of PN(783)10.0 using microkinetic constants from **B**).

To assess the relationship between PN dimensions and transcapillary passage, the classic two compartment pharmacokinetic model (**Figure 4A**) [17] was applied to PN(783)10.0 (**Figure 4B**) and PN(783)4.3 (**Figures 4C**). After injection into normal mice, blood concentrations exhibited an initial fast exponential decay (vascular escape), followed by a slow exponential decay (whole body clearance). The three microscopic rate constants for the two-compartment model are given in **Figures 4B and 4C**. Further details on the two-compartment model are provided in the Supplement with a summary of all pharmacokinetic constants (**Table S1**). The rapid initial fall of PN blood concentration is due to vascular escape, as occurs with fluorescent dextrans [18], [19].

The relative concentrations of PN(783)10.0 in the blood and interstitial compartments using the values from **Figure 4B** are shown in **Figure 4D**. Three pharmacokinetic phases shown are a vascular phase (approximately for 1 h post injection), an interstitial phase at (at 10–25 h), and an enhanced permeability retention (EPR)-based uptake phase (by a tumor) at 48 h. These phases were further examined with fluorescence imaging techniques, SPECT/CT and radioactive organ biodistribution studies below.

### 3. Fluorescent Imaging and Optical Tunability of PN’s

**Figure 5:**
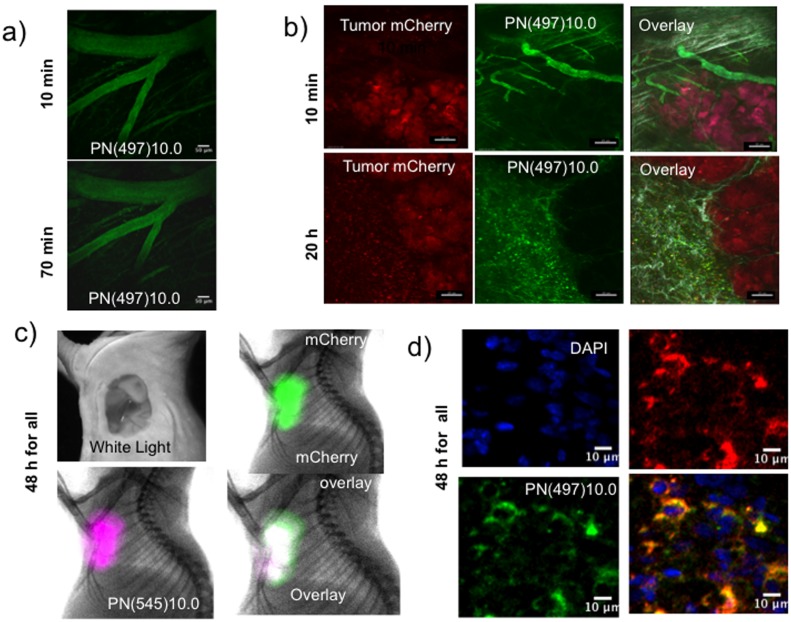
Fluorescent imaging of three pharmacokinetic phases of PN’s with diameters of 10 nm. **A**) Vascular phase, two photon microscopy of brain vasculature of normal mice. Mice underwent a craniotomy and implantation of a transparent window. Vessel intensity drops due vascular escape, but there is no interstitial fluorescence in the brain due to the blood brain barrier. Scale marker = 50 microns. **B**) Intravital confocal microscopy of the vascular and interstitial phases of an mCherry expressing HT-29 xenograft. During the vascular phase (10 min post-injection), vessels are imaged, without interstitial fluorescence. During the interstitial phase (20 h post-injection), interstitial fluorescence is prominent. Scale marker = 20 microns. **C**) Surface fluorescence/X-ray imaging of the tumor retention phase of PN(545)10.0. Shown are the HT-29/mCherry tumor with the skin removed as a white light image, mCherry tumor fluorescence (green), PN(545)10.0 fluorescence (purple) and the green/purple over lay (white). **D**) Confocal microscopy of the tumor retention phase of PN(497)10.0. Shown are a sectioned HT-29 mCherry expressing tumor with nuclei stained blue (DAPI), mCherry tumor cells (red), PN(497)10.0 (green) and a green/red overlay (yellow).

A feature of PN design is that the fluorochrome can be selected to optimize optical properties for different applications. To image the vascular pharmacokinetic phase with two photon microscopy, PN(497)10.0 was used because its fluorescein is readily excited in the two photon mode. (Note that with whole animal surface fluorescence imaging (**Figure 3**), PN’s bearing IR-783 were used because of far greater tissue penetration of their near infrared light.) **Figure 5A** shows intravital fluorescence microscopy of mouse brain capillaries, with the normal blood brain barrier blocking transcapillary passage to the interstitium. To further examine the vascular and interstitial phases, a dorsal skinfold chamber was used for the intravital, two photon microscopy of a mCherry expressing HT-29 tumor (**Figure 5B**). At ten minutes post-injection, PN(497)10.0 (green) was confined to the vasculature at the periphery of the mCherry tumor cells (red). At 20 h post-injection, the PN was seen in the interstitium at the tumor periphery.

The EPR effect with the HT-29 tumor and PN(545)10.0 was demonstrated with surface fluorescence measurements of tumor as shown in **Figure 5C**. With skin removed, an overlay of tumor mCherry (green) and PN(545)10.0 (purple) gave a white superimposition. To determine the cells responsible for the accumulation of PN(783)10.0 seen with surface fluorescence (**Figure 5C**), we employed confocal microscopy of mCherry/HT29 tumor sections (**Figure 5D**). PN(497)10.0 was seen in mCherry/HT29 cells, presumably by fluid phase pinocytosis of rapidly dividing cells, since PEG does not bind known receptors.

### 4. Radiolabeled PN’s for SPECT/CT, Biodistribution and Elimination

**Figure 6:**
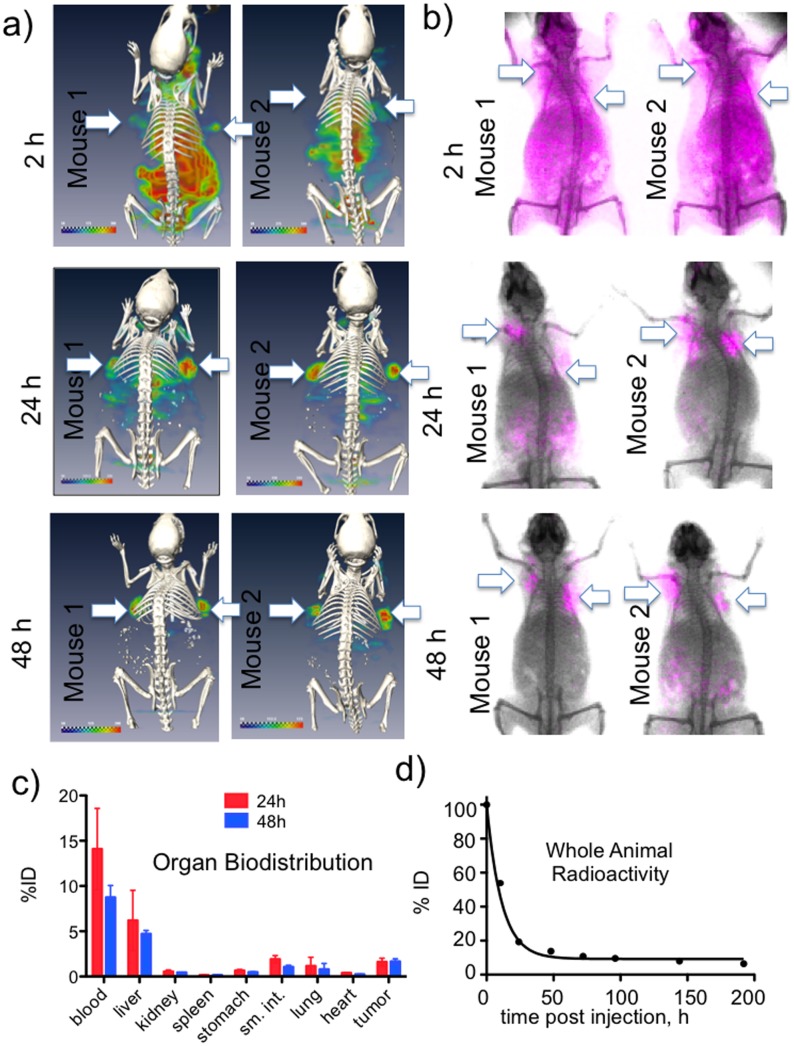
Multimodal imaging of EPR tumor targeting and elimination of PN(783)10.0. **A**) SPECT/CT images of two mice bearing two HT-29 tumors as a function of time after injection. At 2 h post injection, agent is in the blood and interstitium. By 24 h post-injection tumors are becoming apparent as agent is being cleared. At 48 h, labeling is highly tumor selective. **B**) Surface fluorescence imaging of two additional mice bearing the same tumor. By surface fluorescence, as with SPECT, labeling is highly tumor selective at 48 h. **C**) Organ biodistribution was obtained by dissection and ^111^In counting at 24 h and 48 h post-injection. Data are means and standard deviations, n = 5. **D**) A whole animal radioactivity elimination curve. Data are means and standard deviations, with extremely small standard deviations, n = 5.

To demonstrate a multimodal imaging capability, the ability of ^111^In-PN(783)10.0 (or its non-radioactive counterpart, PN(783)10.0) to image the EPR effect of the HT-29 tumor was determined by SPECT/CT (**Figure 6A**) and whole animal surface fluorescence (**Figure 6B**). Initially (2 h post-injection) by SPECT or surface fluorescence, PN(783)10.0 exhibited a broad vascular and interstitial distribution as predicted by the two compartment model which was generated from the time dependence of PN blood concentration (**Figure 4B, 4D**). At 48 h, PN(783)10.0 was seen only in the tumor with SPECT and surface fluorescence, reflecting tomographic slice selection of SPECT and the proximity of the tumor to surface. Consequently radioactive biodistribution studies were performed to quantify tumor and normal organ uptake.

Organ biodistribution studies with ^111^In-PN(783)10.0 at 24 h and 48 h post-injection are shown in **Figure 6C**. At 48 h post injection, 4.71±0.38% of the injected dose (ID) was in the liver, despite the fact that with a diameter of 10 nm this PN is considerably larger than albumin (diameter = 5.4 nm). In addition, only 0.44±0.02% of the injected dose was in the kidney, which typically accumulates high levels of radiolabeled peptides due to renal peptide transporters [20], [21]. Organ concentrations are provided in **Figure S5** in File S1. Whole body radioactivity decreased with a half-life of 7.8 h (**Figure 6D**) and was only 6.41±0.57% of injected dose by 192 h. Thus PN’s show a combination of large size, slow pharmacokinetics and low retention.

### 5. PN Serum Stability

**Figure 7:**
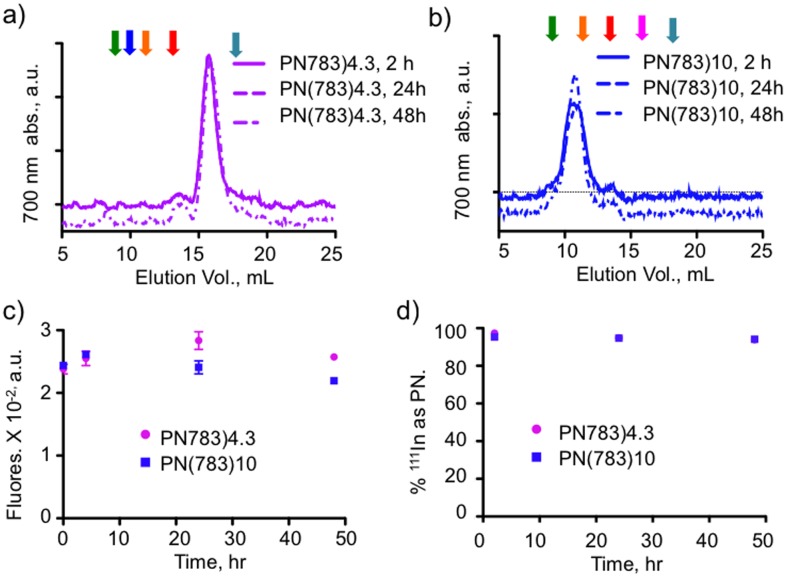
Stability of PN’s in mouse serum. The stability of PN(783)4.3 **A**) or PN(783)10.0 **B**) was examined by incubating nanoprobes for the indicated times and subjecting samples to FPLC. Arrows are the retention times of PN’s from **Figure 2A**, color coded as in that figure. Fluorochrome size is unchanged for both PN’s. **C**) Stability of PN fluorescence. PN(783)4.3 or PN(783)10.0 were incubated as indicated and fluorescence determined. **D**) Stability of ^111^In binding to PN’s. ^111^In-PN(783)4.3 or ^111^In-PN(783)10.0 were incubated as indicated and radioactivity associated with the PN determined by HPLC. Exemplary chromatograms are provided; see **Figure S6** in File S1.

An important requirement of nanoprobes used to study slow passive, pharmacokinetic processes is stability in biological fluids. We therefore examined the stability of PN(783)4.3 and PN(783)10.0 in mouse sera by three techniques: FPLC (size) total probe fluorescence and ^111^In binding over a period of 48 h. As shown by FPLC chromatograms in **Figures 7A and 7B**, PN(783)4.3 and PN(783)10.0 retained their size, consistent post-injection sizes being unchanged from pre-injection sizes (**Figure 2**). Fluorescence of PN(783)4.3 and PN(783)10.0 was also unchanged over this period (**Figure 7C**). Finally, we ^111^In-labeled both PN’s and determined the fraction of radioactivity associated with PN by HPLC as shown in **Figure 7D**. In mouse sera, PN’s exhibit a stable size, a stable fluorescence, and a stable binding of ^111^In.

## Discussion

PEG is often attached to the surfaces of proteins or nanoparticles to obtain improved properties (**Figure 1A**). In contrast PN’s employ a single PEG polymer to surround a central fluorochrome and radiometal bearing peptide, enabling the nanoprobe to be detected by two modalities, and providing the nanoprobe with diameters in nanometer size range.

The PN design allows optical tunability based on fluorochrome selection. Thus, we used PN(783)4.3 and PN(783)10.0 (fluorochrome  =  IR-783, absorption maxima = 783 nm for both PN’s) to demonstrate PEG’s enhancement of elimination by whole animal surface fluorescence imaging (**Figure 3**). On the other hand, with intravital microscopy of capillaries, run in either the two-photon mode (**Figure 5A**) or in the conventional fluorescence mode (**Figure 5B**), PN(497)10.0 was employed (fluorochrome  =  fluorescein, absorption maxima = 497 nm). With intravital microscopy, the higher quantum yields of fluorochromes with shorter absorption and emission maxima may outweigh the greater tissue penetrating properties of near infrared fluorochromes.

PEG attachment significantly increased quantum yield and decreased non-specific binding to cells, relative to the values obtained with unPEGylated fluorochrome-bearing peptides (**Table 1**, columns 6 and 7). However, the magnitude of these benefits varied with the fluorochrome selected. With the (DOTA)Lys-Cys(IR-783) peptide, PEGylation increased the quantum yield from 0.053 to 0.16 or three fold, while with (DOTA)Lys-Cys(Fluorescein) peptide, PEGylation increased quantum yield significantly (p<0.05) but more modestly, from 0.50 to 0.59 or 18%. Previous studies [1] indicated PEGylated, fluorochrome-bearing peptides have absorption spectra in PBS similar to their unstacked absorption spectra in methanol. Hence, a mechanism for the quantum yield improvement afforded by PEGylation is the blockage of fluorochrome/fluorochrome stacking, and fluorochrome self-quenching, that occur in aqueous solutions. It appears that longer wavelength fluorochromes have stronger fluorochrome/fluorochrome stacking interactions, which result in more quenching, which is blocked by PEG and which gives rise to larger increases in quantum yield.

The PN design also offers a pharmacokinetic tunability based on PEG selection, as shown in **Figure 4**, where the pharmacokinetics of PN(783)4.3 and PN(783)10.0 were compared using the classic two-compartment model. The initial, rapid drop in serum fluorescence with both PN’s reflects the high capillary permeability of materials in this size range seen with mice. For example, fluorescent dextrans with molecular weights between 10 and 70 kDa have high capillary permeabilities, reaching peaks of interstitial fluorescence in less than 1 hour post injection [18]. In humans, a specific PN is expected to have substantially slower pharmacokinetics than in mice. Thus PN(783)4.3, which had a 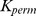 of 1.74 h^−1^ in mice (**Figure 4C**), which can be expressed as a half-life of 23.7 minutes, might be satisfactory as a “long” circulating fluorochrome for intraoperative angiography in humans. ICG, with a blood half-life of 2.5–3 minutes (manufacturer’s package insert) in humans, is currently used for intraoperative fluorescent angiography in neurosurgery [22] or reconstructive surgery [23], [24].

PN’s are nanomaterials that exhibit pharmacokinetics and biodistributions that differ from other fluorochromes and other peptides. As noted, many fluorochromes behave like ICG, binding albumin and undergoing rapid clearance by the hepatobiliary transport system of hepatocytes [25]–[27]. In contrast after injection, PN(783)10.0 underwent a relatively slow vascular permeability (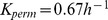) and slow elimination (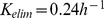), see **Figure 4B** In addition, ^111^In-PN(783)10.0 had a hepatic retention of 4.71±0.38% ID (48 h, **Figure 6C**), though its diameter was significantly larger than albumin (5.4 nm). Whole body radioactivity measurements at 48 h indicated only 13.75±0.74% of ID was retained in the animal (**Figure 6D**), and most of this was in the blood (8.74±1.44% of ID, **Figure 6C**). The presence of ^111^In-PN(783)10.0 in blood results from a combination of nanoprobe transport from the interstitium to the blood, 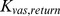 and the slow rate of elimination, 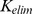, as shown with the two compartment model in **Figure 4A**. Many radiolabeled peptides are substrates for renal peptide transporters, which make the kidney the organ of highest tracer concentration (and organ of dose limiting toxicity) [20], [21], [28]. In contrast, ^111^In-PN(783)10.0 exhibited a renal retention of only 0.44±0.02% of ID (48 h post-injection). ^111^In-PN(783)10.0 is poorly recognized by the cellular transport systems operating *in vivo*, such as the hepatobiliary transport system of hepatocytes which recognizes albumin bound fluorochromes, and renal peptide transporters, which recognize many radiolabeled peptides.

We propose that PN’s consist of a PEG polymer surrounding a fluorochrome and chelate bearing peptide (**Figure 1A**) because of the variety of observations indicating a profound lack of recognition of PN’s in biological systems. These are: (i) PEG reduces nonspecific interactions between fluorochrome bearing peptides with cells **(** **Table 1** **, column 7)**, (ii) PN’s circulate at their PEG determined pre-injection sizes, without binding albumin (**Figures 2D–2F**), a feature that permits pharmacokinetic tunability (**Figure 4**), (iii) after injection, PEG enhances elimination (**Figure 3**) and, (iv) the biodistribution and elimination of ^111^In-PN(783)10.0 (**Figure 6**) shows a lack of recognition by hepatobiliary transport system and renal peptide transporters.

In preliminary clinical trials ICG and fluorescent imaging has been used for the determination of the capillary permeability of breast lesions detected on mammography, indicating a benign or cancerous status [29]. However, a complex ICG infusion protocol [30] was needed to overcome ICG’s short blood half-life, see above. In addition, as shown in **Figure 2G**, ICG can undergo transcapillary passage as the high molecular weight albumin-bound form or as the low molecular weight unbound form. Using PN’s to visualize the capillary permeability, as we did for the HT-29 tumor (**Figure 5B**), is not subject to ambiguities about the molecular form undergoing transcapillary passage. With their pharmacokinetic and optical tunability, PN’s may be superior to ICG for determination of lesion permeability.

To demonstrate an important application for a radiolabeled PN, we imaged tumor EPR effects by SPECT/CT as shown in **Figure 6A**. Long-circulating liposomes and polymer conjugates, approved or in clinical trials, may utilize the EPR effect, in part, for their efficacy [11], [31], [32]. Imaging the EPR effect might lead to improved patient selection for the administration of these often-expensive medications.

In conclusion, PN’s rely on a single long PEG polymer to surround fluorochrome and metal chelate reporters, blocking interactions with cells and biomolecules *in vivo* and *in vitro.* In the vascular phase PN’s might be suitable as controlled blood half-life, intraoperative fluorescent angiographic agents, while in their transition to the interstitial phase used to determine capillary permeability by either fluorescence of SPECT. Finally, the retention of radiolabeled PN’s by tumors might be used to image the EPR effect by SPECT. Thus, the single PEG polymer design of PN’s offers excellent prospects for clinical translation, with uses in multiple clinical applications and multiple imaging modalities.

## Materials and Methods

All animal experiments in this work were approved by the Institutional Review Committee of the Massachusetts General Hospital. Animals were sacrificed by carbon dioxide inhalation. Protected L-amino acids, PyBOP and Rink Amide MBHA resin were from Novabiochem (EMD Biosciences). Other special chemicals were from other sources: DOTA(CO_2_Bu*^t^*)_3_ (Macrocyclics), mPEG-NHS esters (Creative PEGworks or NOF corporation, Japan) IR-783 (Sigma-Aldrich), fluorescein-5-maleimide (Thermo Scientific), and Cy3-maleimide was (Lumiprobe).

The synthesis of PN’s involves three steps: (i) synthesis of the (DOTA)Lys-Cys peptide (**Scheme S1** in File S1), (ii) reaction of thiol reactive fluorochrome to the cysteine thiol (**Schemes S2,S3,S4** in File S1 **)** and, (iii) reaction of an NHS-ester of PEG with variable molecular weight to the lysine side chain **(**Figure S1 and **Schemes S5, S6, S7** in File S1 **)**. Details of each synthesis and radiolabeling (**Scheme S8** in File S1 **)** are given in the supplement.

### PN Characterization

The mass spec of low molecular weight (MW) materials were obtained by MS-ESI Micromass (Waters) and high MW molecules were determined through MALDI-TOF analyses at the Tufts University Core Facility. RP-HPLC (Varian ProStar detector and delivery modules) employed an eluant A (0.1% TFA/water) and eluant B (0.1% TFA and 9.9% water in acetonitrile). Probe size (volume) was determined by FPLC using an ÄKTA Purifier 10 and SuperdexTM 200 10/300GL column (GE Healthcare) with a running buffer of 0.05 M sodium phosphate, 0.1 M NaCl (0.1% Tween, pH 7.2) and flow rate of 0.8 ml/min. Standards (GE Healthcare) were Ferritin, Ribonuclease A, Carbonic Anhydrase, and Conalbumin and Blue Dextran 2000. To obtain probe volumes, Mr (apparent molecular weight based on size exclusion retention) was plotted versus Kav. 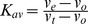, V_t_ = total volume, V_e_ = elution volume, V_o_ = void volume.

### Purity

Peptides were characterized by mass spectroscopy. FPLC was used remove low molecular weight peptide (**Figure S1A** in File S1 **)** and confirm final product purity (**Figure 2**). The mass spec of PN(783)4.3 is shown in **Figure S1B** in File S1.

### Circulating PN’s

20 nmoles of PN(783)4.3, PN(783)6.1, or PN(783)10.0 was injected (IV, tail vein) into nude mice (female; 25–30 g; 6–8 weeks old; nu/nu). At the indicated time, 50 µl of blood was collected with microhematocrit capillary tube (Fisher Scientific) from the tail, and transferred to Eppendorf microcentrifuge tube with anticoagulant (EDTA) coating (Fisher Scientific). Tubes were centrifuged (5000 rpm for 5 min), and the supernatant was injected to the FPLC, a ÄKTA Purifier 10 with SuperdexTM 200 10/300GL column.

### Pharmacokinetics

Groups of 5 nude mice (female; 25–30 g; 6–8 weeks old; nu/nu) were injected (tail vein, IV) with 10 nmole of PN(783)4.3 or PN(783)10.0. 50 µl of blood was collected from tail tip at the indicated times. The blood was processed as above, and diluted (25 µl plasma, 700 µl of PBS). Fluorescence was measured with Cary Eclipse Fluorescence Spectrophotometer, excitation at 765 nm and emission from 790 to 880 nm. The fluorescence intensity at 806 nm was plotted over time, and the data was fit to a biexponential, two phase decay curve. The fast and slow distribution half-life was given by the two phase decay fit with Graphpad Prism software.

### Two Compartment Model

From the two phase decay fit, a biexponential equation for blood concentration as a function of time, 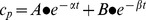, was obtained. By the relation of macro constants and micro constants: 
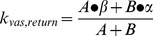
, 
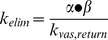
, 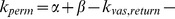 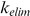, micro constants *k*'s can be obtained, and the half-life was calculated by 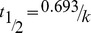, as described in [17]. The curve for interstitium concentration vs. time was fit with MATLAB based on the curve of blood concentration vs. time.

### Whole Animal Surface Fluorescence

A Kodak FX multispectral imaging system was used (Carestream Molecular Imaging, Rochester, NY). Excitation at multiple wavelengths (620, 650, 690, 710, 720, 730, 750 and 760 nm) with the emission at 830 nm was setup for IR-783 spectrum; Excitation at multiple wavelengths (420, 440, 460, 480, 510, 520, 530, and 540 nm) with the emission at 600 nm was setup for Cy3 spectrum; Excitation at multiple wavelengths (450, 470, 510, 520, 530, 540, 550, 570, and 590 nm) with the emission at 700 nm was setup for mCherry; with manufacturer’s software to separate (unmix) the IR-783 spectrum, Cy3 spectrum, or mCherry spectrum from skin autofluorescence and chlorophyll fluorescence from food. X-ray images were taken after fluorescence images. Animals were anesthetized with 2% isoflurane with O_2_ flow (2 l/min) during imaging.

### Tumor Surface Fluorescence (Skin Removed)

The PN(783)10.0 or PN(545)10.0 (10 nmoles, 100 µL) was injected (IV, tail vein), the skin around tumor was removed at 48 h post injection, with tumor visualized as mCherry fluorescence using the Kodak FX.

### Tumor Model

Female nude mice (25–30 g; 6–8 weeks old; nu/nu) were anesthetized with 2% isoflurane/O_2_. HT-29 or mCherry-HT-29 cells were detached, pelleted and 200 µl of cell suspension containing 10^6^ cells in Matrigel (BD Bioscience) was injected subcutaneously into right and left shoulders. Tumors were allowed to grow 5–7 days before experiments. All experiments were approved the MGH committee on animal care. mCherry-HT-29 cells were a gift from Dr. Darshini Kuruppu.

### SPECT/CT

The imaging was performed by Triumph II multimodality imaging system (Gamma Medica Ideas, LLC) comprising XSPECT with four CZT (Cadmium Zink Telluride) detectors and X-O CT with CMOS detector. SPECT data of the ^111^In-labeled compound was acquired for 60 min using 5-pinhole collimators and processed with 3D-OSEM algorithm using 4 subsets and 5 iterations. 3-dimensional CT data was processed with modified Feldkamp software. The processed 3D-images were fused and displayed with VIVID software package installed to the Triumph data management. Animals were under isoflurane anesthesia (1.5%) with O_2_ flow (1.5 l/min) and kept warm during the imaging with a heated animal bed.

### Organ Biodistribution

150 µl of ^111^In-labeled PN(783)10.0 (400 µCi, ∼2 nmole) were injected to tumor-bearing animals by tail vein (IV). 24 h or 48 h later, animals were sacrificed, and tumors, blood, liver, spleen, stomach, kidneys, small intestine, lung, heart, tail, fat, and muscle, were collected. Radioactivity was measured with Perkin Elmer, Wizard2 2480 gamma counter.

### Confocal Imaging

The mCherry-HT-29 tumor sample was collected at 48 h post IV injection with PN(497)10.0, and then cryosectioned with thickness of 5 µm. The tumor section was fixed with 4% PFA, mounted with 90% glycerol/10% PBS (at pH 8.5 for best fluorescein fluorescence), and stained with DAPI. Confocal imaging was performed on a Zeiss LSM510 laser scanning confocal microscope (Zeiss Axiophot, Carl Zeiss, Jena, Germany). A 405 nm diode Laser, 488 nm argon laser, and 561 nm diode laser were used for the excitation of DAPI, fluorescein, and mCherry, respectively. A primary dichroic HFT 405/488/561 was used in combination with an LP420 emission filter for DAPI, BP505-530 for fluorescein, and LP575 for mCherry. Images were analyzed with ImageJ64.

### Brain Vascular Phase Imaging

Craniotomies in C57Bl/6J wildtype mice (from Jackson Laboratory, Bar Harbor, ME. USA, 3–4 months old) were performed as described [33]. Animals were anesthetized using 2% isoflurane in balanced oxygen, and then a 5 mm diameter skull flap was removed. A craniotomy was performed, and the exposed brain area was covered by an 8 mm round glass coverslip, which was sealed to the skull with dental cement. This procedure allowed a transparent window into the mouse brain for use with in vivo microscopy of the cerebrovasculature. Mice were allowed 2–3 weeks for complete recovery after the craniotomy prior to imaging.

Mice were anesthetized with 2% isoflurane in balanced oxygen and secured in a custom stereotaxic frame, which fit into the microscope stage. The cerebrovasculature was imaged using the Olympus FluoView FV1000MPE multiphoton laser-scanning system mounted on an Olympus BX61WI microscope (Olympus, Tokyo, Japan). A DeepSee Mai Tai Ti:sapphire mode-locked laser (Mai Tai; Spectra-Physics, Fremont, CA) produced two photon fluorescence with 800 nm excitation. The vessels were imaged at depth of 45 to 100 µm from the surface of the brain. Two nmoles of PN(497)10.0 was injected with images analyzed Fluoview and ImageJ.

### Imaging the Tumor Interstitium

Dorsal skinfold chamber (DSFC) tumors were grown in female nude mice (nu/nu; 25–30 g; 6–8 weeks old) with modifications from previously published techniques [34], [35]. 10^6^ mCherry-HT-29 tumor cells in matrixgel (BD) were subcutaneously injected in the back of mice ∼1.5 cm left of the dorsal midline approximately halfway from the neck to the tail base. 4 days later, DSFCs were installed in a way that the tumors were centered in the imaging window of the chamber and accessible to longitudinal investigation by MP-IVM. On days 2, 3, and 4 days after tumor DSFC implantation, when tumors were typically 3 mm in diameter, image stacks of tumor tissue were recorded under general anesthesia with Ketamine and Xylazine. Ten nmoles of PN(497)10.0 was injected (IV, tail vein).

Multiphoton excitation was obtained through DeepSee and MaiTai Ti:sapphire lasers (Newport/Spectra-Physics) tuned to 920 and 1000 nm to excite all fluorescent probes used. Stacks of 11 square optical sections with 4 µm z-spacing were acquired every 20 sec on an Ultima IV multiphoton microscope (Prairie Technologies) using a 20X/0.95 NA lens with optical zoom of up to 1x to provide image volumes 30 µm in depth and 200 µm in width. Emitted fluorescence was detected through 460/50, 525/50, 595/50, 660/40 band-pass filters and non-descanned detectors to generate four-color images. Sequences of image stacks were transformed into volume-rendered, time-lapse movies with Imaris software (Bitplane).

### PN Stability

1) Fluorescence: 0.5 nmole of PN(783)4.3 or PN(783)10.0 was incubated in 100 µl of mouse serum (abcam, ab7486) at 37°C for 2 h, 24 h or 48 h. Fluorescence intensity was measured in 1.5 ml cuvette at each time point by diluting with 700 µl of PBS. 2) Size: 10 nmole of PN(783)4.3 or 5 nmole of PN(783)10.0 was incubated in 200 µl of mouse serum at 37°C. 40 µl of mixture was injected into FPLC instrument to measure the size of the nanoprobe at 2 h, 24 h or 48 h. 3) Radiochemical: ^111^In-labeled PN(783)4.3 or ^111^In-labeled PN(783)10.0 was incubated at 37°C in mouse serum for 2 h, 24 h, or 48 h. Radiolabeling stability was determined by HPLC with C18 column.

## Supporting Information

File S1 Supplementary files. Schemes S1 to S8, reactions and conditions used in the synthesis of PN’s. Figure S1, Purification of PN’s by FPLC and PN characterization by mass spectroscopy. Figure S2, Effect of PEG on quantum yields. Figure S3, Dynamic light scattering measurement for PN(783)10.0 and PN(783)11.7. Figure S4, Effect of PEGylation on non-specific binding to cells by single channel FACS. Figure S5, Biodistribution, as organ concentrations, of PN(783)10.0. Corresponding organ biodistributions are provided in Figure 6. Figure S6, HPLC chromatograms of ^111^In-labeled PN’s mouse serum.
(DOCX)
