# Supplementary material for: PEG-Like Nanoprobes: Multimodal, Pharmacokinetically and Optically Tunable Nanomaterials
Source: PLoS One. 2014 Apr 29;9(4):e95406. doi: 10.1371/journal.pone.0095406 (PMC4004541; doi:10.1371/journal.pone.0095406)
Supplement: File S1 — Supplementary files. Schemes S1 to S8, reactions and conditions used in the synthesis of PN’s. Figure S1, Purification of PN’s by FPLC and PN characterization by mass spectroscopy. Figure S2, Effect of PEG on quantum yields. Figure S3, Dynamic light scattering measurement for PN(783)10.0 and PN(783)11.7. Figure S4, Effect of PEGylation on non-specific binding to cells by single channel FACS. Figure S5, Biodistribution, as organ concentrations, of PN(783)10.0. Corresponding organ biodistributions are provided in Figure 6. Figure S6, HPLC chromatograms of 111In-labeled PN’s mouse serum. (DOCX) [file pone.0095406.s001.docx]

Supporting Information for “PEG-like Nanoprobes: Multimodal, Pharmacokinetically and Optically Tunable Nanomaterials”

Yanyan Guo, Hushan Yuan, Natalie M Claudio, Sreekanth Kura, Naomi Shakerdge, Thorsten R. Mempel, Brian J. Bacskai, Lee Josephson*

Supporting Information: Materials and Methods

Protected L-amino acids, PyBOP and Rink Amide MBHA resin were from Novabiochem (EMD Biosciences). Other special chemicals were from other sources: DOTA(CO_2_Bu*^t^*)_3_ (Macrocyclics), mPEG-NHS ester (2-30 kDa from Creative PEGworks; 40 kDa from NOF corporation, Japan), The fluorescent dye IR-783 was purchased from Sigma-Aldrich, fluorescein-5-maleimide was from Thermo Scientific, and Cy3-maleimide was from Lumiprobe. All the other solvents and chemicals were from Sigma-Aldrich.

The synthesis of PN’s involves three steps: (i) synthesis of the (DOTA)Lys-Cys peptide (Scheme 1), (ii) reaction of thiol reactive fluorochrome to the cysteine thiol (Schemes 2,3,4) and, (iii) reaction of an NHS-ester of PEG with variable molecular weight to the lysine side chain (Schemes 5,6,7).

***(i) Synthesis of the (DOTA)Lys-Cys peptide, scheme 1:*** The DOTA(CO_2_*-*Bu*^t^*)_3_-Lys(Boc)-Cys(Trt) peptide was manually synthesized on Rink Amide MBHA resin (0.15 mmol) with an Fmoc/*t-*Bu strategy using a polypropylene 5 mL disposable syringe fitted with a sintered frit. Coupling reactions employed 2 equiv. (relative to resin) of Fmoc-protected amino acid activated in situ with 2 equiv. of PyBOP and 4 equiv. of DiPEA in DMF (10 mL/g resin) for 1-2 hrs. Coupling efficiency was assessed with picrylsulfonic acid. Fmoc groups were removed with a piperidine/DMF solution (1:4) for 4x10 min (10 mL/g resin). The coupling of DOTA was overnight with same equivalent of other reagents. (DOTA)Lys-Cys was released from the solid support with TFA/H_2_O/TIS/EDT 88:2:5:5 (twice, 4 h, 20 mL/g resin). The residue was precipitated and triturated with cold ether. A white solid could be obtained by centrifuge. The solid was purified further by HPLC with column: Higgins Analytical Inc., Clipeus C18 10μm, 250x20 mm; gradient: 20% - 100% B (0.1% TFA and 9.9% water in acetonitrile) in 15min, back to 20%B in 5 min, and isocratic for 5 min. A white powder of compound (DOTA)Lys-Cys was obtained after lyophilization with a yield of 40%. For (DOTA)Lys-Cys, theoretical MW = 634.75, found MW (M+1) =635.57.

***(ii) Synthesis of (DOTA)Lys-Cys(FL) peptides where FL can be IR-783, Cy3 or Fluorescein****.*  See schemes 2-4 below: With all three fluorochromes, the molar ratio of (DOTA)Lys-Cys to fluorochrome 1:1.2. Reaction with IR783 was in DMF, under argon, at room temperature for 15 h, with 6 equiv of DiPEA (Scheme 2 below). The reaction with Cy3-maleimide (scheme 3) or Fluorescein-maleimide (scheme 4) was in DMSO at room temperature for overnight. Products were purified with reverse phase HPLC with a C18 column. The yield of each was around 45% with respect to the starting quantity of fluorochrome. For (DOTA)Lys-Cys(IR-783), theoretical MW = 1325.6, found MW = 1325.8. For (DOTA)Lys-Cys(Cy3), theoretical MW = 1213.6, found MW = 1213.8. For (DOTA)Lys-Cys(Fluorescein), theoretical MW = 1061.4, found (M+1) = 1062.6.

***(iii) Reaction of the (DOTA)Lys-Cys(FL) with NHS esters of PEG, see schemes 5-7 below:*** To a solution of (DOTA)Lys-Cys(fluorochrome) in anhydrous DMSO, was added the solution of PEG-NHS in anhydrous DMSO. The molar ratio of (DOTA)Lys-Cys(fluorochrome) and PEG-NHS was 1:2. After about 6 equiv. of DiPEA was added, the reaction mixture was incubated at room temperature for 7 days. Purification was first by a reverse phase HPLC (C18 column) with gradients as described in ***(i)*** to remove low molecular weight impurities from the synthesis and to obtain an exchange to an aqueous solvent. After lyophilization a second purification was by FPLC, which removed traces of non-PEGylated peptides, see Figure S1. PN’s were concentrated and desalted with a C18 cartridge (Sep-Pak cartridge, Waters, Milford, MA, USA), eluting with acetonitrile and drying by lyophilization. The yield based on starting (DOTA)Lys-Cys(FL), was about 50%.

***Radiolabelling of PN(783)10.0. See scheme 8 below:*** ^111^InCl_3_ (9.43 mCi) (Nordion, Canada) was diluted with HCl (50 μl, 0.05N) into a total volume of 80 μl and was transferred into a conic reaction vial which contained PN(783)/10.0 (20 nmol) in HEPES buffer (1 M, pH 5, 1 ml). The reaction vial was incubated on a preheated heating blot under 70 ^o^C for 45 min while it was shaken every 10 min. Then the vial was cooled down to room temperature in ice water for 5 min. EDTA (70 mM, 100 μl, 7 mmol) was added and well mixed. The solution was stayed at room temperature for 15 min. After the solution was diluted with water (0.1% TFA) (1:10 v/v), the compound was loaded on a C18 cartridge preconditioned with ethanol (1 ml, 0.1% TFA) and water (3 ml, 0.1% TFA) (Strata-X 33u, Polymeric reverse Phase Phenomenex, 30 mg/3 ml, 8B-S100-TBL). The cartridge was washed with water (Millipore, 0.1% HOAc) (2ml) and purged by air with a syringe. The labeled compound was collected by eluting with acetonitrile (0.1% TFA) (0.4 ml) into a new reaction vial. The acetonitrile and TFA were removed by evaporation under N_2_ flow. The final product (4.06 mCi) was reconstituted with PBS buffer for mouse injection. The radioactive product was confirmed to be free of low molecular weight forms of indium by HPLC with cold internal standard with C18 column. (Gradient: 10% B to 100% B in 20 min, back to 10% in 5 min, and isocratic for 5 min; Abs: 783 nm; flow: 5 ml/min; Column: Higgins Analytical Inc. Proto 300 C18 5 μm, 250X10 mm, P/N: CS-2520-C185). RCY: 43%; specific activity: 0.4 Ci / μmole.

***PN and peptide characterization****:* The mass spec of low molecular weights (MW) were obtained by MS-ESI Micromass (Waters) and high MW molecules were determined through MALDI-TOF analyses at the Tufts University Core Facility. RP-HPLC (Varian ProStar detector and delivery modules) employed an eluant A (0.1% TFA /water) and eluant B (0.1% TFA and 9.9% water in acetonitrile). Probe size (volume) was determined by FPLC using an ÄKTA Purifier 10 and SuperdexTM 200 10/300GL column (GE Healthcare) with a running buffer of 0.05 M sodium phosphate, 0.15 M NaCl (0.1% Tween, pH 7.2) and flow rate of 0.8 ml/min. Standards (GE Healthcare) were Ferritin, Ribonuclease A, Carbonic Anhydrase, and Conalbumin and Blue Dextran 2000. To obtain probe volumes, Mr (apparent molecular weight based on size exclusion retention) was plotted versus Kav. $K_{av}=\frac{v_{e}-v_{o}}{v_{t}-v_{o}}$, Vt = total volume, Ve = elution volume, Vo = void volume.

***Purity of materials made:*** The four peptides used (Table 1) were characterized by mass spectroscopy. The use of FPLC to remove low molecular weight peptide is shown in Figure S1A. FPLC’s of the purified PN’s are shown in Figure 2. The mass spec of PN(783)4.3, the peptide (DOTA)Lys(PEG 5 kDa)-Cys(IR-783), is shown in Figure S1B.

***Quantum yield:*** Quantum yields were determined as described [^1^](#_ENREF_1),[^2^](#_ENREF_2). For IR-783 a reference quantum yield of 0.043 was used [^3^](#_ENREF_3); for fluorescein a reference quantum yield was 0.18 [^4^](#_ENREF_4). For Cy3 a reference quantum yield of 0.31 was used (Luminprobe Inc).  For PN(783)’s, excitation was at 730 nm and emission spectra were recorded from 765 nm to 870 nm in PBS and maximum emission used.  For PN(545)’s, excitation was at 515 nm and emission spectra were recorded from 538 nm to 700 nm in PBS and maximum emission used. For PN(497)’s, excitation was at 450 nm and emission spectra were recorded from 475 nm to 620 nm in PBS and maximum emission used.  Absorbance of each probe was adjusted less than 0.1. Measurements were made in triplicate and are expressed as mean ± SD.

***Size by dynamic light scattering:*** Size was obtained on a Nano-ZS Zetasizer (Malvern, Medford, MA) and is expressed as the z-average.

***PN and peptide binding to cells (effect of PEGylation on NSB)*:** HT-29, a human colon carcinoma cell line, was from the American Tissue Culture Collection and maintained according to their instructions. Cells were seeded on 24-well plates at 5X10^5^ cells/well in culture medium (RPMI 1640 with 10% FBS) the day before the assay. The day of assay, medium was removed, wells rinsed twice with DPBS (+Ca, +Mg), and 100 μl of 2% FBS / DPBS (+Ca, +Mg) added. 100 μL of Nanoprobes (2 μM) in DPBS (+Ca, +Mg) was added to cells and incubated for 30min at 37 ^o^C. (Probe concentrations were determined spectrophotometrically (783 nm, extinction coefficient of 314 471 cm^-1^ M^-1^ for IR-783; 497 nm, extinction coefficient of 68 000 cm^-1^ M^-1^ for Fluorescein; 545 nm, extinction coefficient of 150 000 cm^-1^ M^-1^ for Cy3). Cells were detached by Trypsin/EDTA and assayed for fluorescence by FACS (BD 7 laser LSR2 for nanoprobes with IR-783; BD 3 laser LSR2 for nanoprobes with Fluorescein or Cy3).

***Circulating form of PN’s:*** 20 nmoles of PN(783)4.3, PN(783)6.1, or PN(783)10.0 was injected (IV, tail vein) into nude mice (female; 25-30 g; 6-8 weeks old; nu/nu). At the indicated time, 50 μl of blood was collected with microhematocrit capillary tube (Fisher Scientific) from the tail, and transferred to Eppendorf microcentrifuge tube with anticoagulant (EDTA) coating (Fisher Scientific). Tubes were centrifuged (5000 rpm for 5 min), and the supernatant was injected to the FPLC, a ÄKTA Purifier 10 with SuperdexTM 200 10/300GL column.

***PN pharmacokinetics*:** Groups of 5 nude mice (female; 25-30g; 6-8 weeks old; nu/nu) were injected (tail vein, IV) with 10 nmole of PN(783)4.3 or PN(783)10.0. 50 μl of blood was collected from tail tip at the indicated times. The blood was processed as above, and diluted (25 μl plasma, 700 μl of PBS). Fluorescence was measured with Cary Eclipse Fluorescence Spectrophotometer, excitation at 765 nm and emission from 790 to 880 nm. The fluorescence intensity at 806 nm was plotted over time, and the data was fit with two-phase decay curve. The fast and slow distribution half-life was given by the two-phase decay fit with Graphpad Prism software.

***Two compartment model:*** From the two-phase decay fit, a biexponential equation for blood concentration as a function of time, $c_{p}=A\bullet e^{-\alpha t}+B\bullet e^{-\beta t}$, was obtained. By the relation of macro constants and micro constants, $k_{vas,return}=\frac{A\bullet\beta+B\bullet\alpha}{A+B}$, $k_{e\lim}=\frac{\alpha\bullet\beta}{k_{vas,return}}$, $k_{perm}=\alpha+\beta-k_{vas,return}-k_{e\lim}$, micro constants *k*'s can be obtained, and the half-life was calculated by $t_{\frac{1}{2}}=\frac{0.693}{k}$, as described in [^5^](#_ENREF_5). The curve for interstitium concentration vs. time was fit with MATLAB based on the curve of blood concentration *vs.* time.

***Whole animal surface fluorescence imaging*:** All animal experiments in this work were approved by the Institutional Review Committee of the Massachusetts General Hospital. A Kodak FX multispectral imaging system was used (Carestream Molecular Imaging, Rochester, NY). Excitation at multiple wavelengths (620, 650, 690, 710, 720, 730, 750 and 760 nm) with the emission at 830 nm was setup for IR-783 spectrum; Excitation at multiple wavelengths (420, 440, 460, 480, 510, 520, 530, and 540 nm) with the emission at 600 nm was setup for Cy3 spectrum; Excitation at multiple wavelengths (450, 470, 510, 520, 530, 540, 550, 570, and 590 nm) with the emission at 700 nm was setup for mCherry; with manufacturer’s software to separate (unmix) the IR-783 spectrum, Cy3 spectrum, or mCherry spectrum from skin autofluorescence and chlorophyll fluorescence from food. X-ray images were taken after fluorescence images. Animals were anesthetized with 2% isoflurane with O_2_ flow (2 l/min) during imaging.

***Tumor surface fluorescence (skin removed):*** The PN(783)10.0 or PN(545)10.0 (10 nmoles, 100 µL) was injected (IV, tail vein), the skin around tumor was removed at 48 h post injection, with tumor visualized as mCherry fluorescence using the Kodak FX.

***HT-29 or mCherry-HT-29 tumor model*:** Female nude mice (25-30g; 6-8 weeks old; nu/nu) were anesthetized with 2% isoflurane/O_2_. HT-29 or mCherry-HT-29 cells were detached, pelleted and 200µl of cell suspension containing 10^6^ cells in Matrigel (BD Bioscience) was injected subcutaneously into right and left shoulders. Tumors were allowed to grow 5-7 days before experiments. All experiments were approved the MGH committee on animal care. mCherry-HT-29 cells were a gift from Dr. Darshini Kuruppu.

***SPECT/CT*:** The imaging was performed by Triumph II multimodality imaging system (Gamma Medica Ideas, LLC) comprising XSPECT with four CZT (Cadmium Zink Telluride) detectors and X-O CT with CMOS detector. SPECT data of the ^111^In-labeled compound was acquired for 60 min using 5-pinhole collimators and processed with 3D-OSEM algorithm using 4 subsets and 5 iterations. 3-dimensional CT data was processed with modified Feldkamp software. The processed 3D-images were fused and displayed with VIVID software package installed to the Triumph data management. Animals were under isoflurane anesthesia (1.5%) with O_2_ flow (1.5 l/min) and kept warm during the imaging with a heated animal bed.

***Organ biodistribution of ^111^In-PN(783)10.0:*** 150 µl of ^111^In-labeled PN(783)10.0 (400 µCi, ~2 nmole) were injected to tumor-bearing animals by tail vein (IV). 24 h or 48 h later, animals were sacrificed, and tumors, blood, liver, spleen, stomach, kidneys, small intestine, lung, heart, tail, fat, and muscle, were collected. Radioactivity was measured with Perkin Elmer, Wizard2 2480 gamma counter.

***Confocal imaging:*** The mCherry-HT-29 tumor sample was collected at 48 h post IV injection with PN(497)10.0, and then cryosectioned with thickness of 5 µm. The tumor section was fixed with 4% PFA, mounted with 90% glycerol/10% PBS (at pH 8.5 for best fluorescein fluorescence), and stained with DAPI. Confocal imaging was performed on a Zeiss LSM510 laser scanning confocal microscope (Zeiss Axiophot, Carl Zeiss, Jena, Germany). A 405 nm diode Laser, 488 nm argon laser, and 561 nm diode laser were used for the excitation of DAPI, fluorescein, and mCherry, respectively. A primary dichroic HFT 405/488/561 was used in combination with an LP420 emission filter for DAPI, BP505-530 for fluorescein, and LP575 for mCherry. Images were analyzed with ImageJ64.

***Brain vascular phase imaging (angiography):*** Craniotomies in C57Bl/6J wildtype mice (from Jackson Laboratory, Bar Harbor, ME. USA, 3-4 months old) were performed with minor modifications [^6^](#_ENREF_6). To summarize, animals were anesthetized using 2% isoflurane in balanced oxygen, and then a 5 mm diameter skull flap was removed. A craniotomy was performed, and the exposed brain area was covered by a 8 mm round glass coverslip, which was sealed to the skull with dental cement [^6^](#_ENREF_6),[^7^](#_ENREF_7) This procedure allowed a transparent window into the mouse brain for use with in vivo microscopy of the cerebrovasculature. Mice were allowed 2-3 weeks for complete recovery after the craniotomy prior to imaging.

For imaging, mice were anesthetized with 2% isoflurane in balanced oxygen and secured in a custom stereotaxic frame, which fit into the microscope stage. The cerebrovasculature was imaged using the Olympus FluoView FV1000MPE multiphoton laser-scanning system mounted on an Olympus BX61WI microscope (Olympus, Tokyo, Japan). A DeepSee Mai Tai Ti:sapphire mode-locked laser (Mai Tai; Spectra-Physics, Fremont, CA) produced two-photon fluorescence with 800 nm excitation. The vessels were imaged at depth of 45 to 100μm from the surface of the brain.

2 nmole of PN(497)/10.0 probe (300-400µl) was injected retro-orbital into the anesthetized mouse. A time course was taken for up to 70 minutes post injection. Images were acquired using the Fluoview software and analyzed using ImageJ.

***Imaging tumor interstium*:** Dorsal skinfold chamber (DSFC) tumors were grown in female nude mice (nu/nu; 25-30g; 6-8 weeks old) with modifications from previously published techniques [^8^](#_ENREF_8),[^9^](#_ENREF_9). 10^6^ mCherry-HT-29 tumor cells in matrixgel (BD) were subcutaneously injected in the back of mice ~ 1.5 cm left of the dorsal midline approximately halfway from the neck to the tail base. 4 days later, DSFCs were installed in a way that the tumors were centered in the imaging window of the chamber and accessible to longitudinal investigation by MP-IVM. On days 2, 3, and 4 days after tumor DSFC implantation, when tumors were typically 3 mm in diameter, image stacks of tumor tissue were recorded under general anesthesia with Ketamine and Xylazine. 100 µl (10 nmole) of PN(497)10.0 was injected (IV, tail vein).

Multiphoton excitation was obtained through DeepSee and MaiTai Ti:sapphire lasers (Newport/Spectra-Physics) tuned to 920 and 1000 nm to excite all fluorescent probes used. Stacks of 11 square optical sections with 4 μm z-spacing were acquired every 20 sec on an Ultima IV multiphoton microscope (Prairie Technologies) using a 20X/0.95 NA lens with optical zoom of up to 1x to provide image volumes 30 μm in depth and 200 μm in width. Emitted fluorescence was detected through 460/50, 525/50, 595/50, 660/40 band-pass filters and non-descanned detectors to generate four-color images. Sequences of image stacks were transformed into volume-rendered, time-lapse movies with Imaris software (Bitplane).

***Stability tests:*** 1) Fluorescence: 0.5 nmole of PN(783)4.3 or PN(783)10.0 was incubated in 100 μl of mouse serum (abcam, ab7486) at 37 ^o^C for 2h, 24h or 48h. Fluorescence intensity was measured in 1.5 ml cuvette at each time point by diluting with 700 μl of PBS. 2) Size stability: 10 nmole of PN(783)4.3 or 5 nmole of PN(783)10.0 was incubated in 200 μl of mouse serum at 37 ^o^C. 40 μl of mixture was injected into FPLC instrument to measure the size of the nanoprobe at 2h, 24h or 48h. 3) Radiolabeling stability: ^111^In-labeled PN(783)4.3 or ^111^In-labeled PN(783)10 was incubated at 37 ^o^C in mouse serum for 2h, 24h, or 48h. Radiolabeling stability was determined by HPLC. Gradients: 10%B to 80%B in 15 min, then back to 10%B in 5 min, and continue 10%B for 5 min. Flow: 5 ml/min. Detection at 783 nm. Column: Higgins Analytical Inc. Proto 300 C18 5 μm, 250 X 10 mm, P/N: CS-2520-C185. The free ^111^In was eluted around 3.5 min and the ^111^In-labeled nanoprobes were eluted around 11 min.

Supporting Information, Results

**Purification and Characterization of PN’s**

***
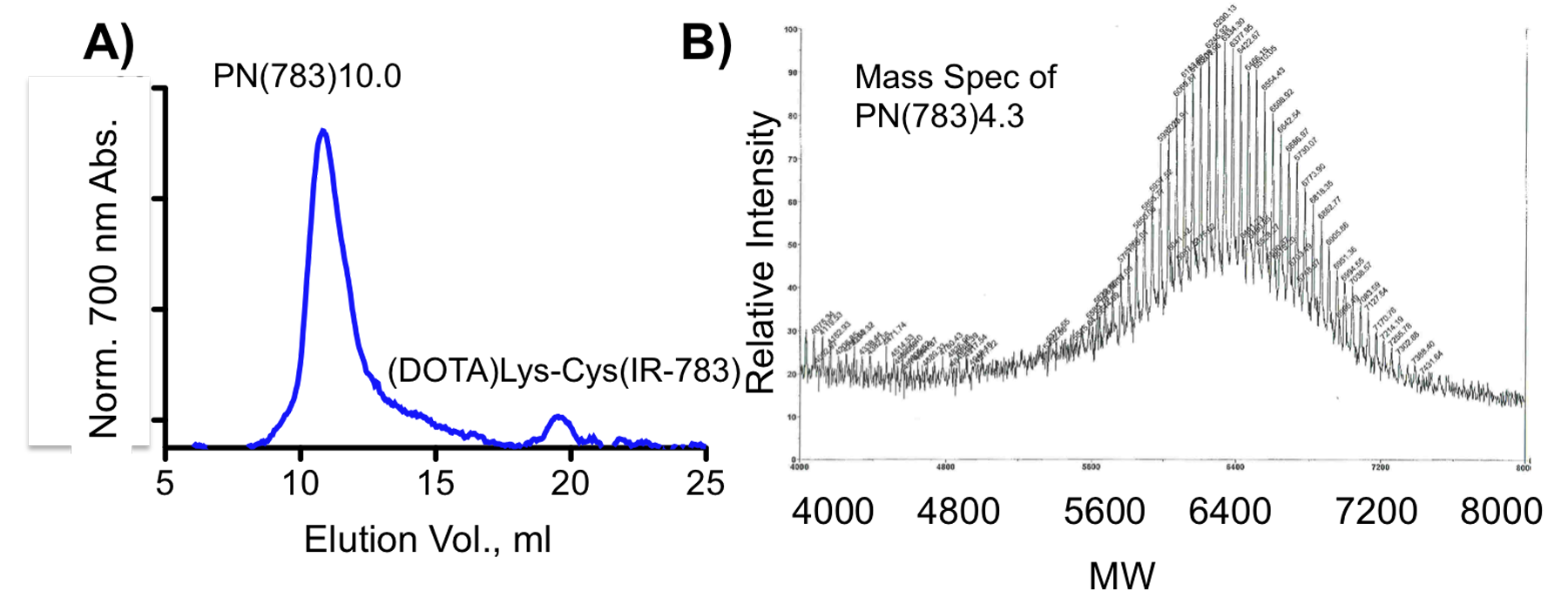
***

**Figure S1: Purification of PN’s by FPLC and PN characterization by mass spectroscopy.** A) FPLC chromatogram of purification of PN(783)10.0 by removal of the low molecular weight (DOTA)Lys-Cys(IR-783) peptide which is used in PN synthesis. FPLC’s of pure PN’s are shown in Figure 2. B) MALDI-TOF Mass spectroscopy of pure PN(783)4.3 made by reaction of (DOTA)Lys-Cys(IR-783) with the 5 kDa PEG-NHS. Note the absence of species at 4800 to 5200 Da, expected if there was PEG contamination.

**The Effect of PEG on Quantum Yields**

**
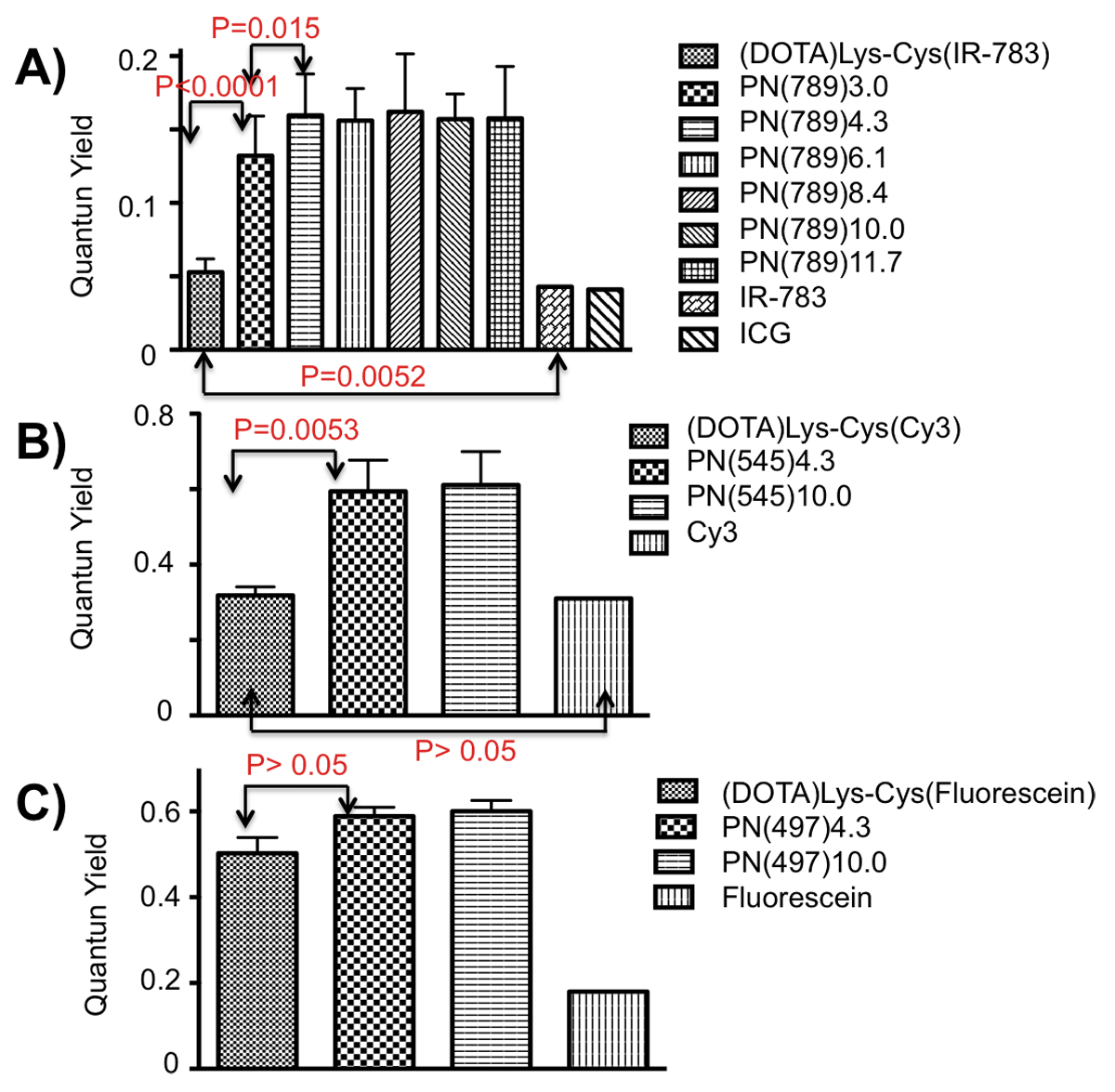
**

**Figure S2: Effect of PEG on Quantum Yields.**  A) Quantum yields are shown for PN(783)’s made with different PEG’s. B) Quantum yields for PN(545)’s made with different PEG’s. C) Quantum yields with PN(497)’s made with different PEG’s. As excitation maxima go up quantum yields go down. Quantum yields are always improved by PEGylation but the degree of improvement varies.

**Light scattering Determination of PN Size**

**
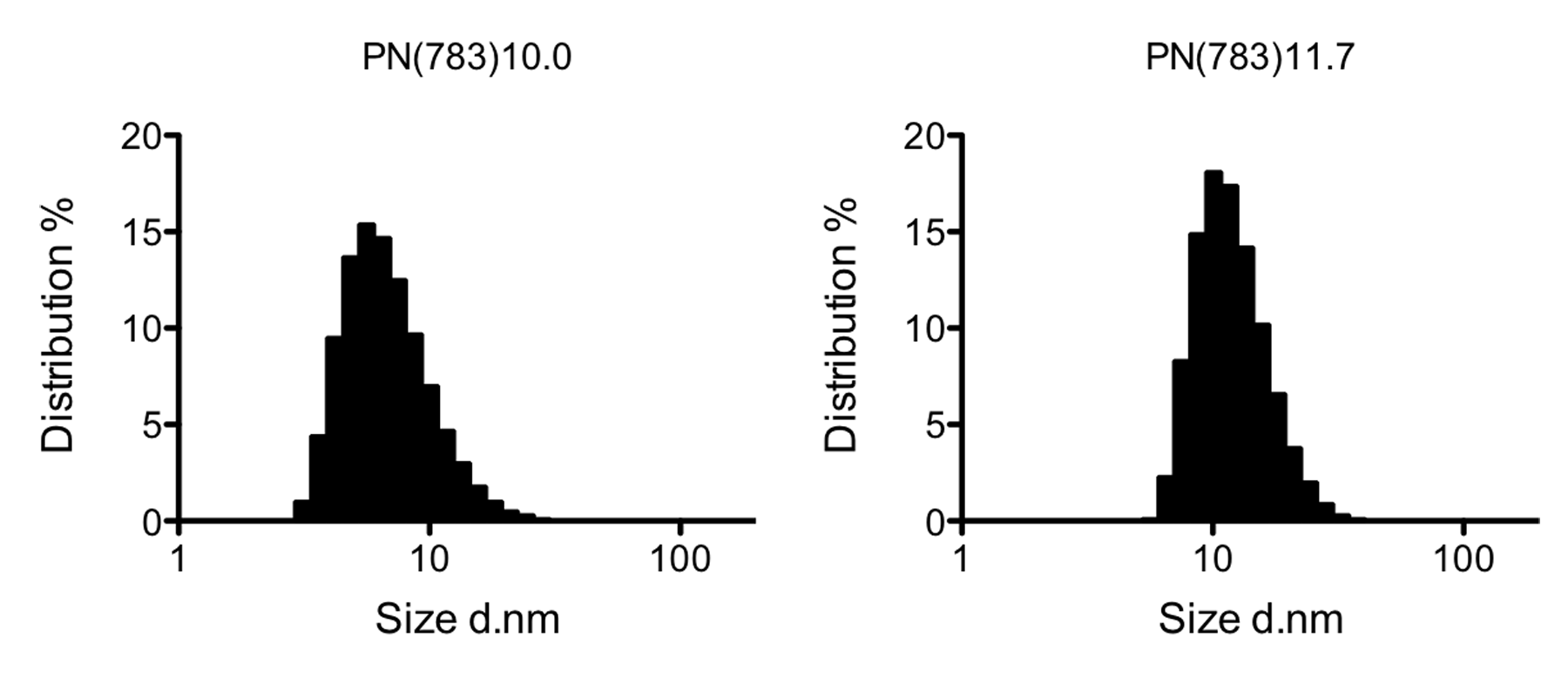
**

**Figure S3**: Dynamic light scattering measurement for PN(783)10.0 and PN(783)11.7.

**The effect of PEG on Nonspecific Binding to HT-29 Cells**

**
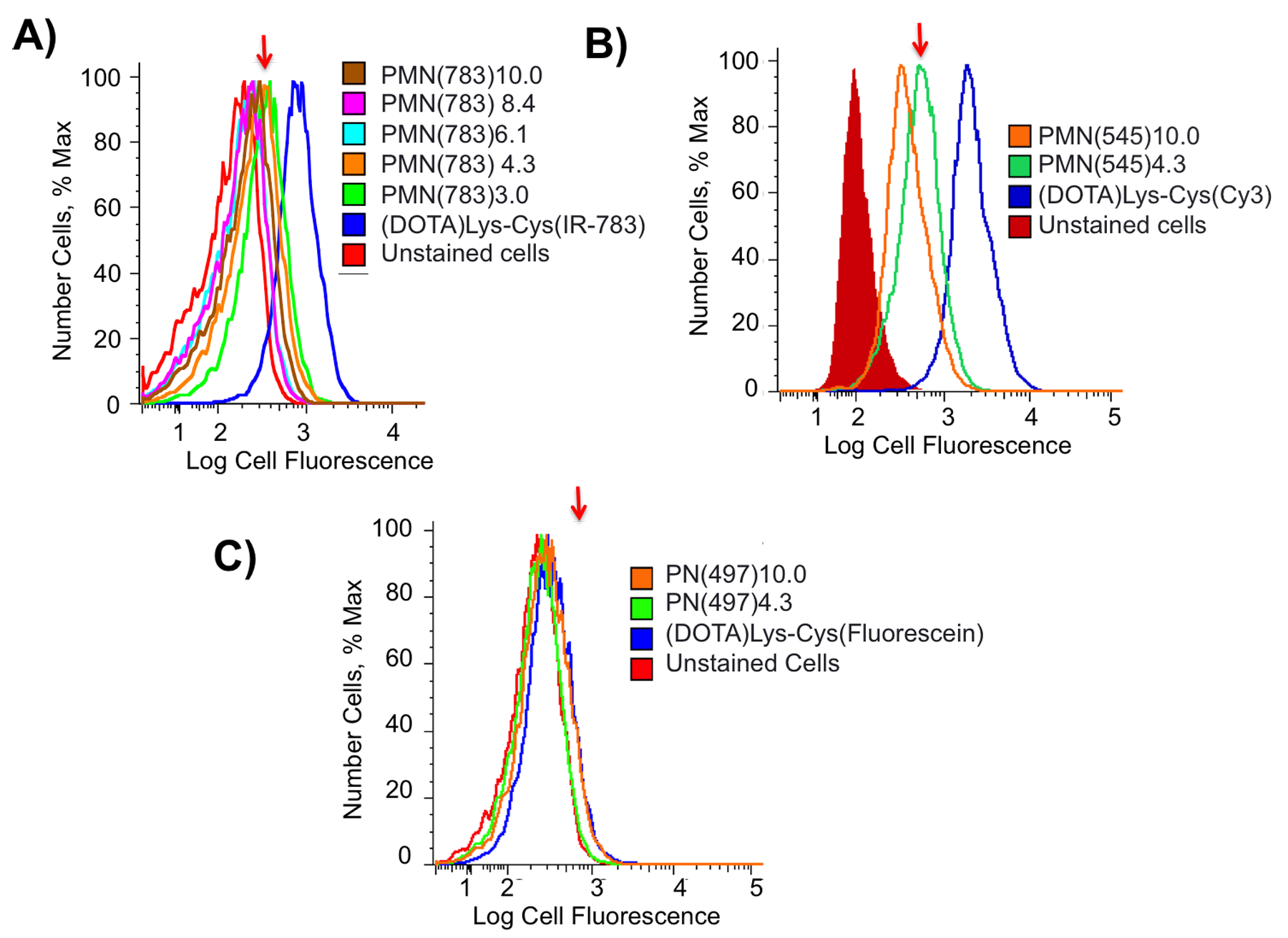
**

**Figure S4: Effect of PEGylation on non-specific binding to cells by single channel FACS.** A) The (DOTA)Lys-Cys(IR-783) peptide binds cells but PEGylated versions have greatly reduced binding. The percent of cells with fluorescence higher than unstained cell is given in Table 1. B) The (DOTA)Lys-Cys(Cy3) peptide binds to cells but PEGylated versions have greatly reduced binding. C) The (DOTA)Lys-Cys(Fluorescein) peptide binds cells very weakly so PEG does not reduce binding. Arrows indicates the cutoff for unstained cells.


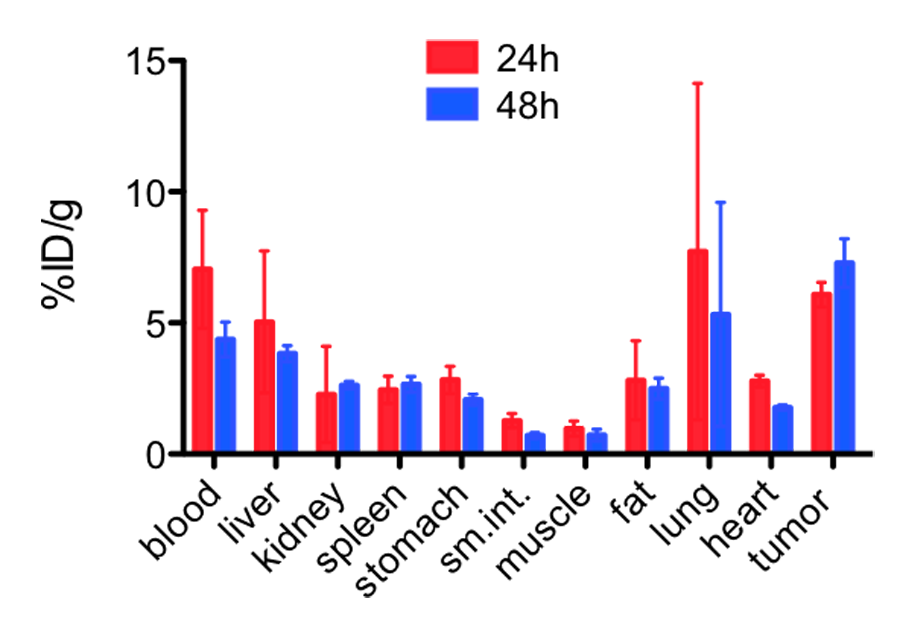


**Figure S5: Biodistribution, as organ concentrations, of PN(783)10.0.** Corresponding organ biodistributions are provided in Figure 6.

**
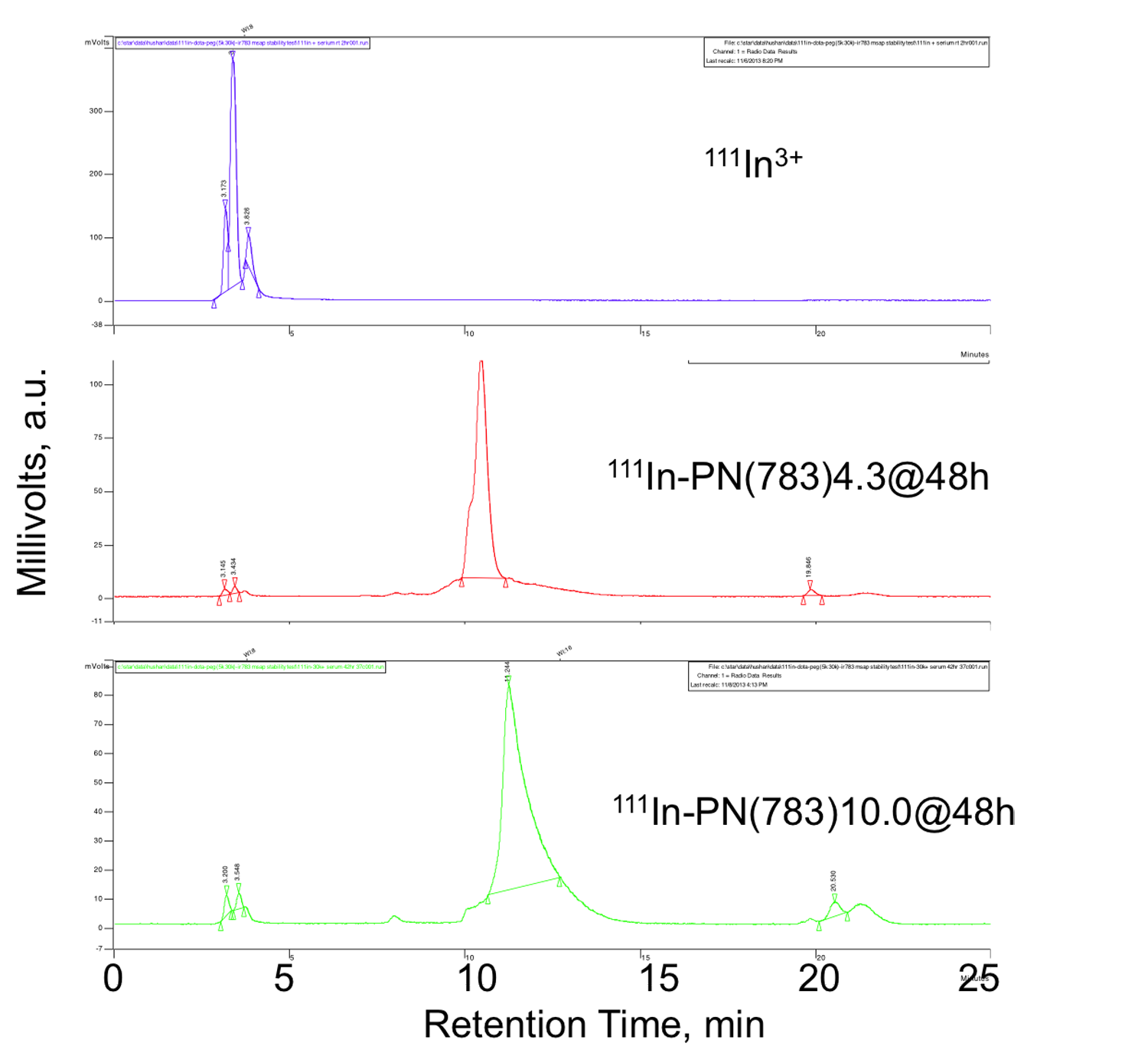
**

**Figure S6: HPLC chromatograms of ^111^In-labeled PN’s mouse serum.** Exemplary chromatograms used to generated stability data in Figure 7D are shown.

**Two compartment model, Figure 3.**

Serum fluorescence data from Figure 3B and Figure 3C was analyzed using the two-compartment model as described [^5^](#_ENREF_5). Data were first fit to biexponential equation, yielding values of Alpha, Beta (apparent decay constants) and values of A and B as shown in equations 1 and 2 below.

General Biexpponential Equation: $c_{p}=A\bullet e^{-\alpha t}+B\bullet e^{-\beta t}$

Equation 1, PN(783)10: $c_{p}=1039.5e^{-1.361t}+595.2e^{-0.09903t}$

Equation 2, PN(783)4.3: $c_{p}=112.8e^{-5.301t}+161e^{-0.425t}$

By the relation of macro constants and micro constants, $k_{vas,return}=\frac{A\bullet\beta+B\bullet\alpha}{A+B}$, $k_{e\lim}=\frac{\alpha\bullet\beta}{k_{vas,return}}$, $k_{perm}=\alpha+\beta-k_{vas,return}-k_{e\lim}$, micro constants k's can be obtained, and the half-life was calculated by $t_{\frac{1}{2}}=\frac{0.693}{k}$, see above.

Table S1: Summary of constants for PN(783)4.3 and PN(783)10.0 obtained with the two-compartment model, Figure 4.

| Constant | PN(783)4.3  rate | PN(783)4.3  half-life | PN(783)10.0  rate | PN(783)10.0  half-life |
| --- | --- | --- | --- | --- |
| Alpha | 5.30 h^-1^ | 0.13 h | 1.36 h^-1^ | 0.51 h |
| Beta | 0.42 h^-1^ | 1.64 | 0.099 h^-1^ | 7.0 h |
| ^111^In-PN(783)10.0 elimination[[1]](#footnote-1) |  |  |  | 7.8 h |
| k perm | 1.74 h^-1^ | 0.40 h | 0.67 h-1 | 1.02 h |
| k vas return | 3.30 h^-1^ | 0.20h | 0.56 h^-1^ | 1.23 h |
| k elim | 0.68 h^-1^ | 1.01h | 0.24 h^-1^ | 4.2 h |

See figure 6D.

**REFERENCES FOR SUPPORTING INFORMATION**

1. Demas, J.N. & Crosby, G.A. Measurement of photoluminescence quantum yields. Review. *Journal of Physical Chemistry* 1971, *75*, 991-1024.

2. Shao, F., Yuan, H., Josephson, L., Weissleder, R. & Hilderbrand, S.A. Facile Synthesis of Monofunctional Pentamethine Carbocyanine Fluorophores. *Dyes and pigments : an international journal* 2011, *90*, 119-122.

3. Li, C., Greenwood, T.R., Bhujwalla, Z.M. & Glunde, K. Synthesis and characterization of glucosamine-bound near-infrared probes for optical imaging. *Organic letters* 2006, *8*, 3623-3626.

4. Sjoback, R., Nygren, J. & Kubista, M. Absorption and florescence propertie of fluorescein. *Spectrohimica Acta Part A* 1995, *51*, L7-L21.

5. Rosenbaum, S.E. *Basic Pharmacokinetics and Pharmacodynamics: An Integrated Textbook and Computer Simulations*, (John Wiley and Sons, Hoboken, NJ. , 2011).

6. Skoch, J., Hickey, G.A., Kajdasz, S.T., Hyman, B.T. & Bacskai, B.J. In vivo imaging of amyloid-beta deposits in mouse brain with multiphoton microscopy. *Methods in molecular biology (Clifton, N.J.)* 2005, *299*, 349-363.

7. Spires-Jones, T.L., de Calignon, A., Meyer-Luehmann, M., Bacskai, B.J. & Hyman, B.T. Monitoring protein aggregation and toxicity in Alzheimer's disease mouse models using in vivo imaging. *Methods (San Diego, Calif.)* 2011, *53*, 201-207.

8. Fukumura, D.*, et al.* Tumor induction of VEGF promoter activity in stromal cells. *Cell* 1998, *94*, 715-725.

9. Marangoni, F.*, et al.* The transcription factor NFAT exhibits signal memory during serial T cell interactions with antigen-presenting cells. *Immunity* 2013, *38*, 237-249.

1. [↑](#footnote-ref-1)
